# Genome wide association study identifies novel single nucleotide polymorphic loci and candidate genes involved in soybean sudden death syndrome resistance

**DOI:** 10.1371/journal.pone.0212071

**Published:** 2019-02-26

**Authors:** Sivakumar Swaminathan, Anindya Das, Teshale Assefa, Joshua M. Knight, Amilton Ferreira Da Silva, João P. S. Carvalho, Glen L. Hartman, Xiaoqiu Huang, Leonor F. Leandro, Silvia R. Cianzio, Madan K. Bhattacharyya

**Affiliations:** 1 Department of Agronomy, Iowa State University, Ames, Iowa, United States of America; 2 Department of Computer Science, Iowa State University, Ames, Iowa, United States of America; 3 USDA and Department of Crop Sciences, University of Illinois, Urbana, Illinois, United States of America; 4 Department of Plant Pathology and Microbiology, Iowa State University, Ames, Iowa, United States of America; Agriculture and Agri-Food Canada, CANADA

## Abstract

*Fusarium virguliforme* is a soil borne root pathogen that causes sudden death syndrome (SDS) in soybean [*Glycine max* (L.) Merrill]. Once the fungus invades the root xylem tissues, the pathogen secretes toxins that cause chlorosis and necrosis in foliar tissues leading to defoliation, flower and pod drop and eventually death of plants. Resistance to *F*. *virguliforme* in soybean is partial and governed by over 80 quantitative trait loci (QTL). We have conducted genome-wide association study (GWAS) for a group of 254 plant introductions lines using a panel of approximately 30,000 SNPs and identified 19 single nucleotide polymorphic loci (SNPL) that are associated with 14 genomic regions encoding foliar SDS and eight SNPL associated with seven genomic regions for root rot resistance. Of the identified 27 SNPL, six SNPL for foliar SDS resistance and two SNPL for root rot resistance co-mapped to previously identified QTL for SDS resistance. This study identified 13 SNPL associated with eight novel genomic regions containing foliar SDS resistance genes and six SNPL with five novel regions for root-rot resistance. This study identified five genes carrying nonsynonymous mutations: (i) three of which mapped to previously identified QTL for foliar SDS resistance and (ii) two mapped to two novel regions containing root rot resistance genes. Of the three genes mapped to QTL for foliar SDS resistance genes, two encode LRR-receptors and third one encodes a novel protein with unknown function. Of the two genes governing root rot resistance, *Glyma*.*01g222900*.*1* encodes a soybean-specific LEA protein and *Glyma*.*10g058700*.*1* encodes a heparan-alpha-glucosaminide N-acetyltransferase. In the LEA protein, a conserved serine residue was substituted with asparagine; and in the heparan-alpha-glucosaminide N-acetyltransferase, a conserved histidine residue was substituted with an arginine residue. Such changes are expected to alter functions of these two proteins regulated through phosphorylation. The five genes with nonsynonymous mutations could be considered candidate SDS resistance genes and should be suitable molecular markers for breeding SDS resistance in soybean. The study also reports desirable plant introduction lines and novel genomic regions for enhancing SDS resistance in soybean.

## Introduction

Soybean [*Glycine max* (L.) Merrill] oil and protein meal accounts for approximately 25% and 65% of the world consumption, respectively [1]. Sudden death syndrome (SDS) is the second most damaging soybean disease in the U.S. with an estimated soybean yield suppression valued to $0.6 billion in 2014 [2]. The disease has two components: (i) foliar SDS or leaf scorch and (ii) root rot. In North America as well as in South America, *Fusarium virguliforme* O’Donnell and T. Aoki (formerly *F*. *solani* (Mart.) Sacc. f. sp. *glycines*) causes SDS. It has only one mating type and is asexually propagated [3]. In South America, *F*. *virguliforme* and *F*. *tucumaniae* cause SDS. *F*. *tucumaniae* has both mating types and is sexually propagated [3]. In addition to these two major SDS pathogens, in South America *F*. *crassistipitatum* and *F*. *brasiliense* are also reported to cause SDS in soybean [4–6].

The *F*. *virguliforme* causes root rot and root necrosis [7]. Upon penetration through the cortex into the root xylem, the pathogen secretes toxins that cause chlorotic and necrotic interveinal foliar symptoms [7–11]. The name ‘sudden death syndrome’ derives from the observations that following infection and toxin translocation, leaves of normal-appearing plants suddenly develop interveinal chlorosis and necrosis and plants die prematurely [12–14].

Management options for controlling SDS are limited [15, 16]. Growing of SDS resistant varieties is the most effective method of protecting the crop from this fungal pathogen [17, 18]. Unfortunately, SDS resistance is partial and encoded by a large number genes, each contributing small effect [17, 19–31]. Furthermore, epistatic interactions among SDS resistance QTL result in even a more complex inheritance for the SDS resistance [32, 33].

Most of the SDS resistance QTL have been mapped using the progenies of bi-parental crosses [17, 19–32]. In recent years, genome wide association studies (GWAS) have identified 30 single nucleotide polymorphic loci (SNPL) linked to foliar SDS resistance [33–35]. Some of the SNPL were co-mapped to SDS resistance QTL that were previously identified through studies of bi-parental crosses [31, 33–35]. The population structures used in the GWAS have been different. Chang et al. (2016) [34] used a group of ancestral lines for mapping the SDS resistance loci. Zhang et al. (2015) [33] used a set of PI lines; and Wen et al. (2014) [35] a group of advanced experimental lines and released cultivars to conduct GWAS in identifying SNPL associated with SDS resistance.

The majority of the QTL for SDS resistance or SNPL identified are for foliar SDS. *F*. *virguliforme* is a root pathogen and causes damages to the infected roots [36]. Limited studies have identified soybean genotypes that carry both foliar SDS and root rot resistance [15, 32, 37–39]. Therefore, more soybean germplasm with both foliar SDS and root rot resistance may be identified for providing robust protection against *F*. *virguliforme*. The objectives of the study were therefore to identify (i) genetic materials with novel sources of foliar SDS and root rot resistance genes, and (ii) genomic regions and genes involved in both foliar SDS and root rot resistance. The phenotypic evaluation of 254 preselected PI lines [40, 41] for both foliar SDS and root rot was conducted under growth-chamber conditions using a mixture of *F*. *virguliforme* Mont-1 isolate and two highly aggressive isolates collected in Iowa. We have identified ten PI lines that are resistant to both foliar SDS and root rot symptoms and should be suitable resources for breeding soybean cultivars with robust SDS resistance. GWAS revealed 13 SNPL associated with eight novel genomic regions containing foliar SDS resistance genes and six SNPL with five novel regions for root-rot resistance. We have identified five candidate genes for foliar SDS and root rot resistances that can be potential molecular markers for introgressing SDS resistance into soybean cultivars.

## Materials and methods

### Plant material

The 254 PI lines studied here were selected from a collection of over 6,000 PI lines based on their preliminary responses to *F*. *virguliforme* Mont-1 isolate in greenhouse, recorded by Hartman lab, University of Illinois, Urbana [40, 41]. Seeds of the 254 accessions were kindly provided by the USDA-ARS-GRIN National Genetic Resources Collection lab (S1 Table). The maturity group (MG) of the accessions ranged from MG 000 to MG X.

### *F*. *virguliforme* isolates

In this research the responses of the PI lines to a mixture of three *F*. *virguliforme* isolates, Mont-1, Scott F2I11a and Clinton 1B were studied in growth chambers located at the Department of Agronomy, Iowa State University. The highly aggressive *F*. *virguliforme* isolates, Clinton-1B and Scott-F2I11a, were collected from Clinton and Scott Counties, Iowa, respectively [42, 43]. The single-spore derived isolates Clinton-1B (LL0059) and Scott F2I11a (LL0063) are being stored and maintained at the Leandro lab. The *F*. *virguliforme* Mont-1 isolate was collected from Monticello, Illinois. The *F*. *virguliforme* Mont-1 (FSG1) isolate has been widely used by the soybean research community as an aggressive isolate and its genome has been sequenced [4, 15, 40, 44–48].

### Inoculum preparation

The methods of inoculum preparation and infection were described earlier [49]. Each isolate was grown on potato dextrose agar (PDA) (13 g Difco PDA/L) amended with antibiotics (0.150 g/L of streptomycin sulfate, and 0.15 g/L of chlortetracycline hydrochloride) for 6 weeks at room temperature (19–23°C) under dark conditions.

Sterile white sorghum [*Sorghum bicolor* (L.) Moench] seeds were soaked in distilled water for 24 h in quart mason jars (500 g seeds/jar). After the water was drained off, the sorghum filled jars were autoclaved twice, 1 h in each time. Sterile sorghum kernels of each jar were inoculated with ten mycelial plugs (7 mm in diameter) of the individual *F*. *virguliforme* isolate. Jars were incubated at room temperature (21 ± 2° C) for one month under continuous fluorescent light (40 W) and shaken daily for 1 to 2 min to ensure uniform fungal growth. After a month, the infested sorghum kernels were placed on a tray under a fume hood for 24 h to dry. Then the inocula were stored at 4° C and used in all five experiments to avoid any variation resulting from the individual batches of inocula.

The DNA concentration of the *F*. *virguliforme* isolate in individual inoculum was determined by qPCR. The DNA concentration ranged from 25 to 30 ng /mg of infested dried sorghum kernels [49]. The three inocula prepared for three *F*. *virguliforme* isolates were combined in equal proportions based on the DNA concentration of individual isolate. The thoroughly mixed inoculum prepared from three isolates was then added to pasteurized soil and sand (1:2) mix at a proportion of 1:20 :: inoculum:soil and sand mix. The inoculum was mixed thoroughly with the soil and sand mix by hand.

### Phenotyping

The seeds of the soybean lines were planted in inoculum mixed soil-sand mix in Styrofoam cups (240 mL). Three seeds of individual soybean genotypes were placed on the surface of inoculum mixed soil-sand mix and additional 30 mL inoculum mixed soil:sand mixture was added to cover the seeds. The cups were randomized in a growth chamber. Cups were watered once daily. The seedlings were grown at 23 ± 1 °C (day time) and 16 ± 1 °C (night time) with 16 h light and 8 h dark periods. Light intensity was maintained at 300 μmol photons m^−2^s^−1^.

Five weeks after planting, foliar disease score of each plant was recorded using a modified scale (S1 Fig) of the one originally described by Hartman et al. (2004) [44]: 1, no foliar symptoms observed; 1.5, leaves showing a few chlorotic specks (1–5% foliage affected); 2, leaves showing slight yellowing and/or chlorotic flecks or blotches (6–10% foliage affected); 2.5, leaves showing big chlorotic flecks or blotches (11–20% foliage affected); 3, leaves showing interveinal chlorosis (21–30% foliage affected); 3.5, leaves showing interveinal chlorosis spread throughout the plant (31–40% foliage affected); 4, leaves start to fold (cupping of leaves), with slight necrosis (41–50% foliage affected); 4.5, leaves with necrosis along the >2 cm sectors along the leaf margin (51–60% foliage affected); 5, necrosis along the entire margin of the leaves (61–70% foliage affected); 5.5, heavy necrosis and cupping of leaves (71–80% foliage affected); 6, most of the leaf area necrotic and the leaves being heavily rolled and/or irregular in shape (81–90% foliage affected); 6.5, most of leaf area necrotic and dry (>90% foliage affected); 7, leaf drop resulting in defoliated plants (S1 Fig). On the basis of foliar disease scores (FDS), PIs were classified as highly resistant (HR; FDS <1.51); resistant (R; FDS 1.51–2.00); moderately resistant (MR; FDS 2.01–2.50); susceptible (S; FDS 2.51–3.00); or highly susceptible (HS; FDS >3.00) [44].

Thirty-seven days after planting, plants were carefully removed from the cups and roots were washed with warm tap water. Root rot (%) was evaluated as root areas showing dark brown to black discolorations, visually assessed on a percentage scale from 0 to 100 with an increment of 5% of the total root area (S2 Fig) [14].

### Statistical analysis

The model for the statistical analyses for foliar SDS scores and root rot (%) data was,

$${\text{Y}}_{\text{ij}}=\mu +{\text{g}}_{\text{i}}+{\text{b}}_{\text{j}}+{\text{e}}_{\text{ij}},$$

where, Y_ij_ is the observed phenotype of the ith genotype in jth block or experiment, μ is the overall population mean, g_i_ is the genetic effect of the ith genotype, b_j_ is the effect of the jth block, and e_ij_ is the effect of experimental error. In each growth chamber experiment, three cups carrying a total of nine plants for each of the 254 PI lines and control lines were randomized. The mean disease scores or extent of root rot (%) were calculated from the plants of the three cups of each PI line. The experiment was repeated five times.

The mean phenotypic data of each genotype from each of the five experiments were subjected to analysis of variance (ANOVA) in a randomized block design by considering each experiment as a block, and tested for homogeneity of variances among experiments using the Levene’s test in the R package car [50]. Fisher’s protected least significant difference (LSD) test was used to compare means at *p* ≤ 0.05 using the R package agricolae [51]. Estimation of variance components for foliar SDS scores and root rot were determined using the R package. The histograms and fitted lines describing the distribution of foliar disease scores and extent of root rot (%) were generated using R software (package fBasics).

Broad sense heritability (*H*^*2*^) estimates for FDS and root rot (%) were estimated using the formula, *H*^*2*^ = σ_g_^2^/(σ_g_^2^ + σ_e_^2^), where, σ_g_^2^is the genotypic variance; σ_e_^2^ is the error variance; and σ_g_^2^ + σ_e_^2^ is phenotypic variance (σ_p_^2^).

### Genotyping and quality control

The SNP dataset for the PIs was reported earlier by Song et al. (2013) [52], and it was downloaded from SoyBase (http://www.soybase.org/; 2018 version). Imputation of some data points was conducted using BEAGLE version 3.3.1. Of the total SNPs 42,168, accessible from SoyBase for the association panel, 59 SNPs were unanchored to the reference genome and were removed from further analysis. Individual SNP markers with missing rate larger than 10% were omitted from the analysis and only 31,506 SNPs were used in GWAS. The statistically significant SNPL were not originated from the imputed SNPL.

### Genome-wide association study

The phenotypic data were analyzed using the best linear unbiased prediction (BLUP) program and the R lem4 package to reduce the effect of experimental variation [53]. GWAS was performed with the mixed linear model (MLM) using genome assessment and prediction integrated tools (GAPIT) in the R package [54, 55]. The most stringent approach between the false discovery rate (FDR) at *p* < 0.05 and empirical significant level at *p* < 0.001 was used to determine the threshold of significance for SNP-trait association, as described earlier [33]. A 1,000 permutations of genome-wide association was conducted to assess the empirical significant of SNPs [33]. SoyBase (ww.soybase.org; Glyma.Wm82.a2 Gmax2.0) was searched to find out additional information on SNPL and their associated candidate SDS resistance genes.

## Results

### Infection of PI lines with *F*. *virguliforme* isolates revealed novel PI lines with foliar SDS and root rot resistance

**Fig 1:**
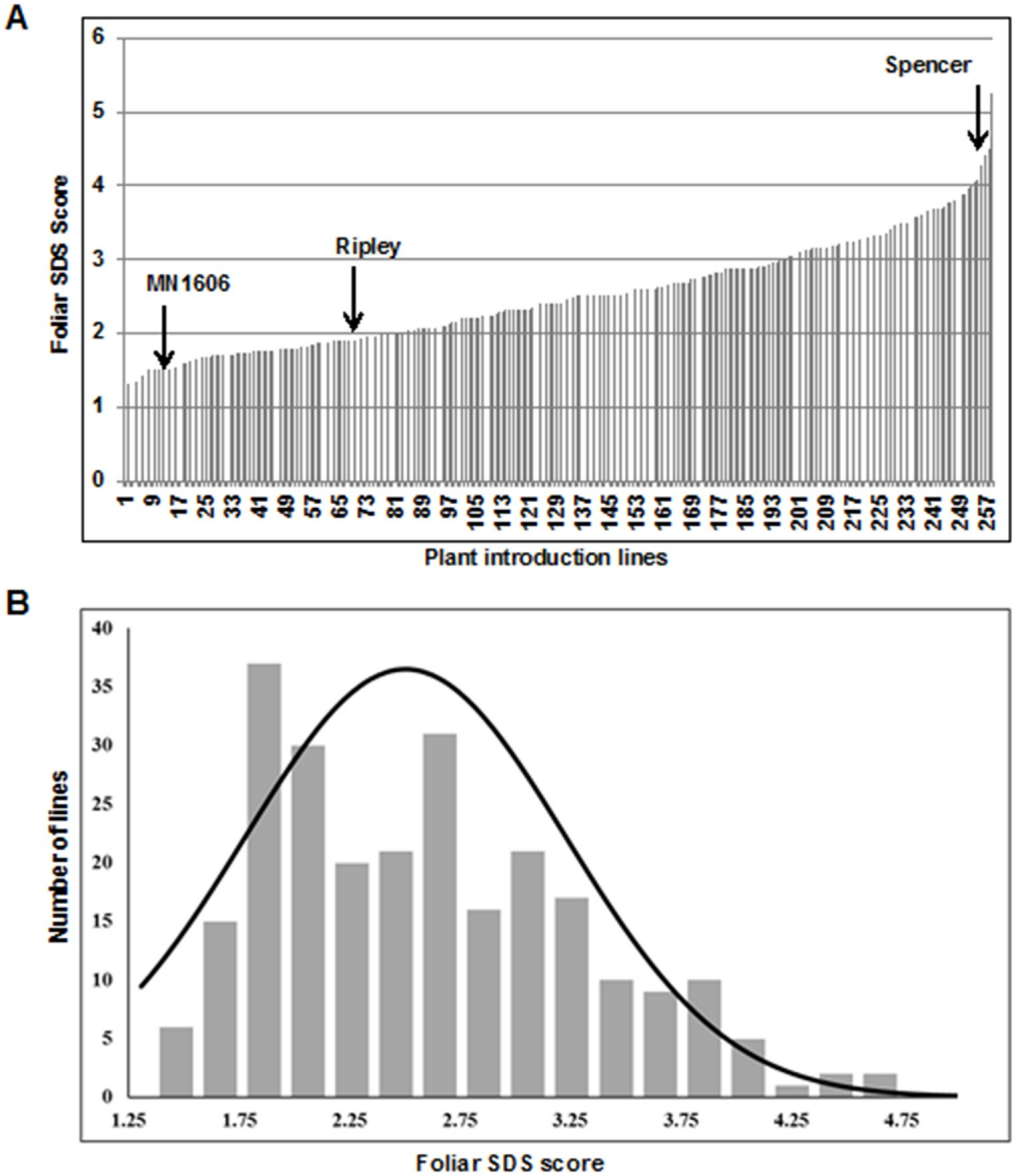
Foliar responses of 254 PI lines to *F*. *virguliforme*. (A) The foliar disease symptoms were scored 35 days following infection with *F*. *virguliforme*. Arrows indicate the disease scores of the SDS resistant cultivars, MN1606 and Ripley, and susceptible cultivar, Spencer. Phenotypic evaluation was conducted five times, each with three replications. The values are means of fifteen biological replications. (B) Distribution of foliar SDS scores among the 254 PI that are lines presented in (A).

**Fig 2:**
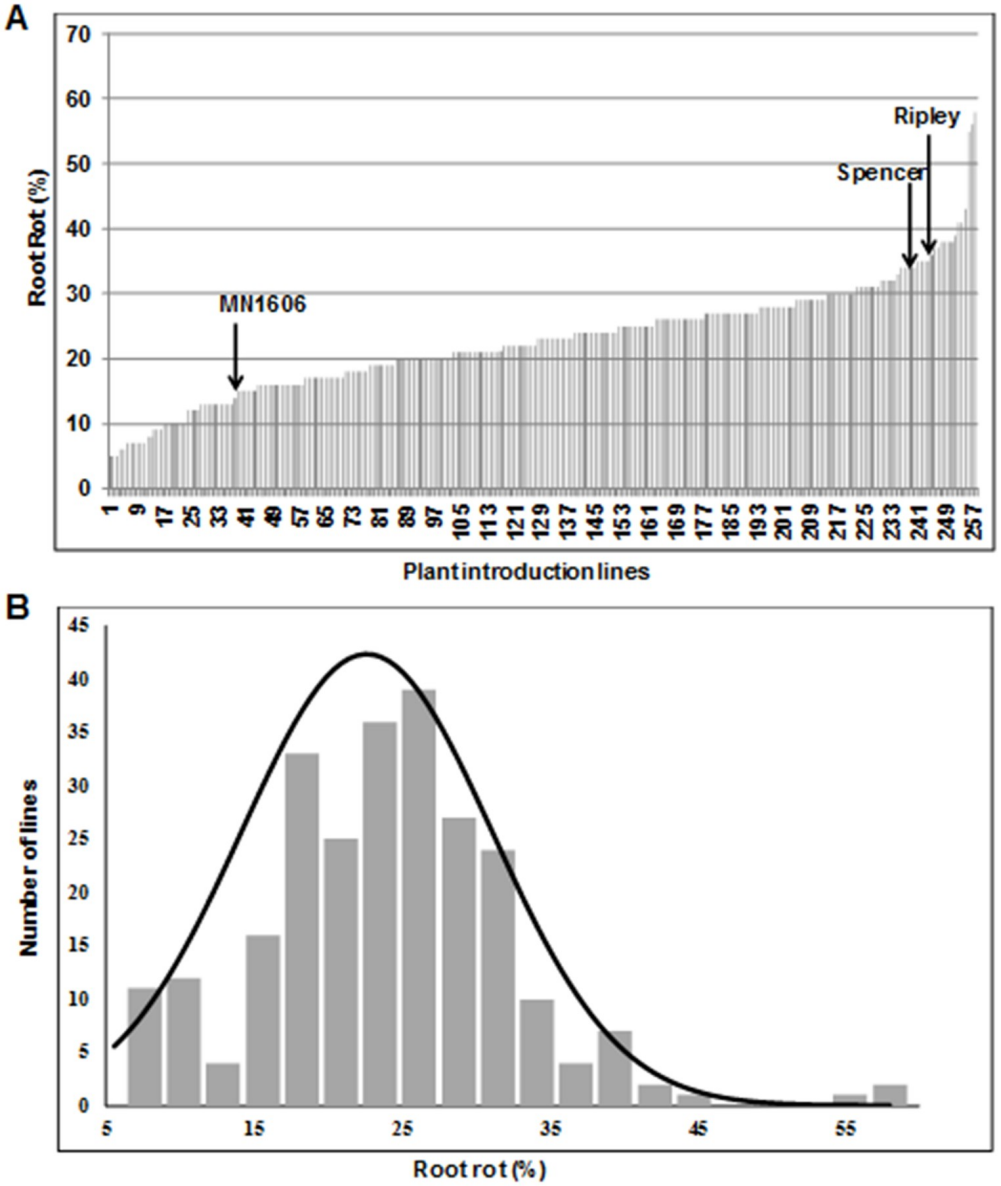
Root responses of 254 PI lines to *F*. *virguliforme*. (A) The root rot symptoms were scored 37 days following infection with *F*. *virguliforme*. Arrows indicate the disease scores of the SDS resistant cultivars, MN1606 and Ripley, and susceptible cultivar, Spencer. Phenotypic evaluation was conducted five times, each with three replications. The values are means of fifteen biological replications. (B) Distribution of root rot symptoms among the 254 PI lines that are presented in (A).

The 254 PI lines considered for this study were selected from a collection of over 6,000 PI lines based on their preliminary resistant responses to *F*. *virguliforme* Mont-1 isolate [40, 41]. The lines were evaluated for leaf and root responses against a mixture of three highly aggressive *F*. *virguliforme* isolates including Mont-1, Scott F2I11a and Clinton 1B, under growth chamber conditions. The lines were also evaluated for 31,506 single nucleotide polymorphism loci (SNPL). To study the structure of this sub-population of 254 PI lines, a neighbor joining (NJ) tree and principal component analysis (PCA) of the lines were conducted using Tassel 5.2.33 program on 31,506 single nucleotide polymorphisms (SNPs) distributed across the soybean genome [52]. The NJ tree of SNPs revealed six groups (S3 Fig). Both the NJ tree and PCA indicated a possible weak association between genotypes and geographic origin (S1 Table, S4A Fig). The PCA was also conducted for maturity groups (MGs) of the PI lines. No apparent association between MGs and genotypes of the PI lines was observed (S4B Fig). Similarly, foliar as well as root responses of the lines collected using a published protocol [49] to a mixture of three aggressive *F*. *virguliforme* isolates, Mont-1, Scott F2I11a and Clinton 1B were used to study the population structure. Both foliar and root responses of the 254 lines were randomly distributed across the population (S4C and S4D Fig). Although the foliar responses of the lines to *F*. *virguliforme* Mont-1 isolate was not normal (S5 Fig), the responses of the lines to the mixture of three *F*. *virguliforme* isolates including Mont-1 exhibited a normal distribution (*p* < 0.05) (Figs 1 and 2; S6 Fig). The Levene’s test of homogeneity of variance suggested that variation among the five experiments were homogenous for foliar as well as for root rot symptoms (*p* > 0.05). ANOVA revealed significant differences (*p* < 0.05) for both foliar SDS and root rot symptoms among the genotypes.

Mean foliar SDS score of the resistant check MN1606 was 1.53, significantly different (*p* < 0.05), from that (4.5) of the susceptible check, Spencer (Fig 1). For root rot scores, the resistant check MN1606 exhibited 15% root rot, which is significantly (*p* < 0.01) less than that (34%) of the susceptible check, Spencer (Fig 2). Among the 254 PI lines, seven were highly foliar SDS resistant with foliar SDS scores of <1.5, while 71 were resistant with scores ranging from 1.50–2.00, and 61 PIs were highly susceptible with foliar SDS scores of >3.00 (S1 Table). Among the 254 PI lines, 23 lines showed <10% root rot and six showed >40% root rot (S1 Table).

**Fig 3:**
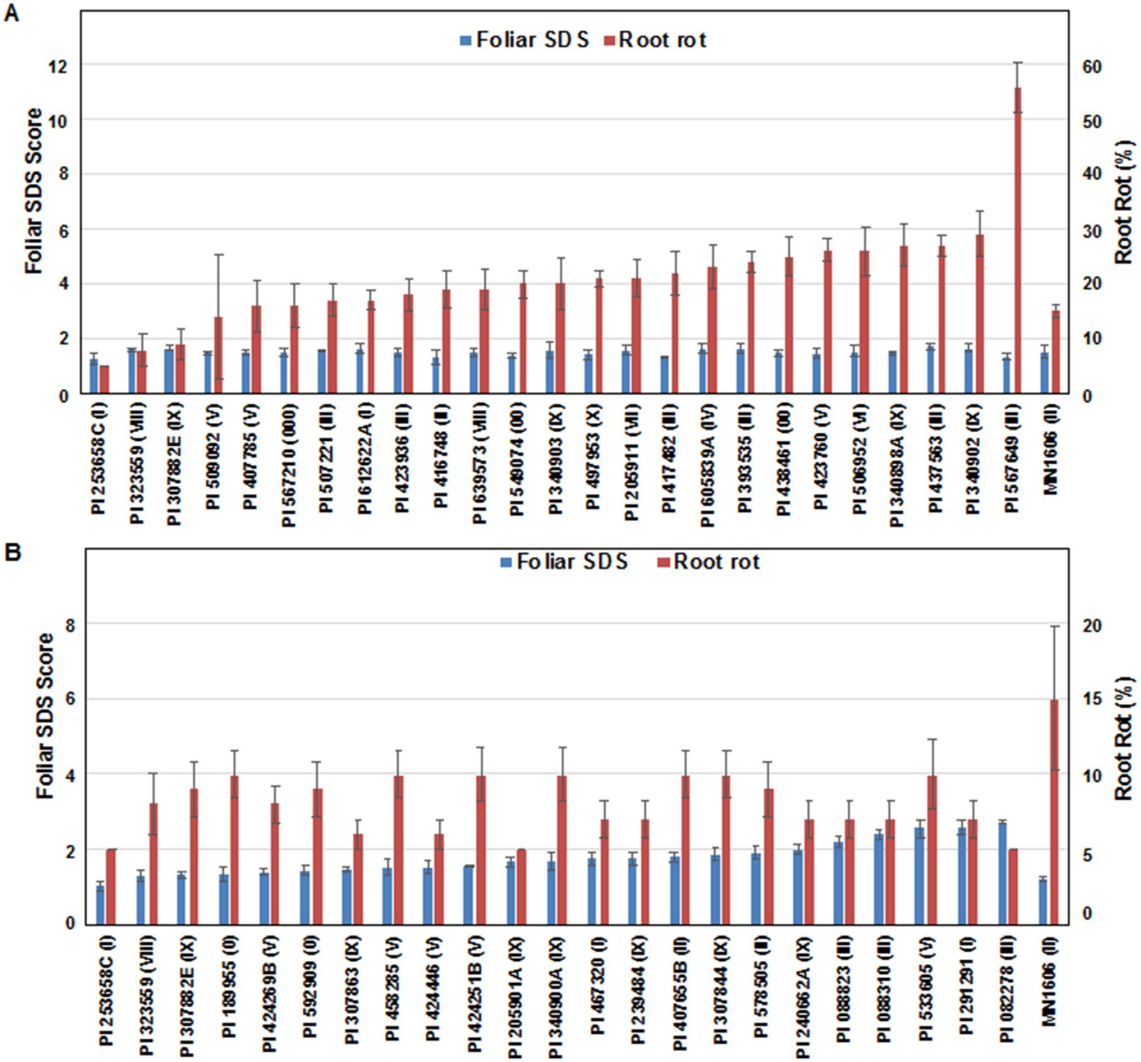
Segregation of foliar and root responses of 25 PI lines *F*. *virguliforme*. (A) Root responses of 25 most foliar SDS resistant PI lines to *F*. *virguliforme*. (B) Foliar responses of 25 most root rot resistant PI lines to *F*. *virguliforme*. The values are means and standard errors calculated from five independent experiments. The Scott-Knott statistical significance difference of the PI lines are provided in S1 Table.

A weak but significant association was observed between foliar SDS scores and root rot (*r* = 0.19; *p* < 0.01). The 25 most foliar SDS resistant PI lines are presented in Fig 3A to show that the root rot resistances of these lines are variable ranging from 5 to 56% root rot. Similarly, 23 PI lines with root rot scores less than 10% exhibited variation in the foliar SDS scores, which ranged from 1.26 to 3.42 (Fig 3B). We observed that 10 lines showed foliar disease scores < 2 and 10% or less root rot (S1 Table). These lines could be suitable for breeding SDS resistant soybean cultivars.

### Genome wide association study (GWAS) revealed novel SNPL and candidate genes for SDS resistance

The broad sense heritability values calculated were 0.90 and 0.77 for foliar SDS and root rot symptoms, respectively. The high heritability values suggest that the variation observed in the phenotypic data among the PI lines was less influenced by experimental variation and phenotypic data collected in the growth chamber were suitable for conducting GWAS to detect the genetic loci associated with the SDS resistance. GWAS was conducted using the mixed linear model (MLM) [54, 55]. The threshold of significance for SNP-trait association was determined by the levels of significance for false discovery rate (FDR) at *p* < 0.05 and empirical significant level at *p* < 0.001 as described in Materials and Methods [33].

**Fig 4:**
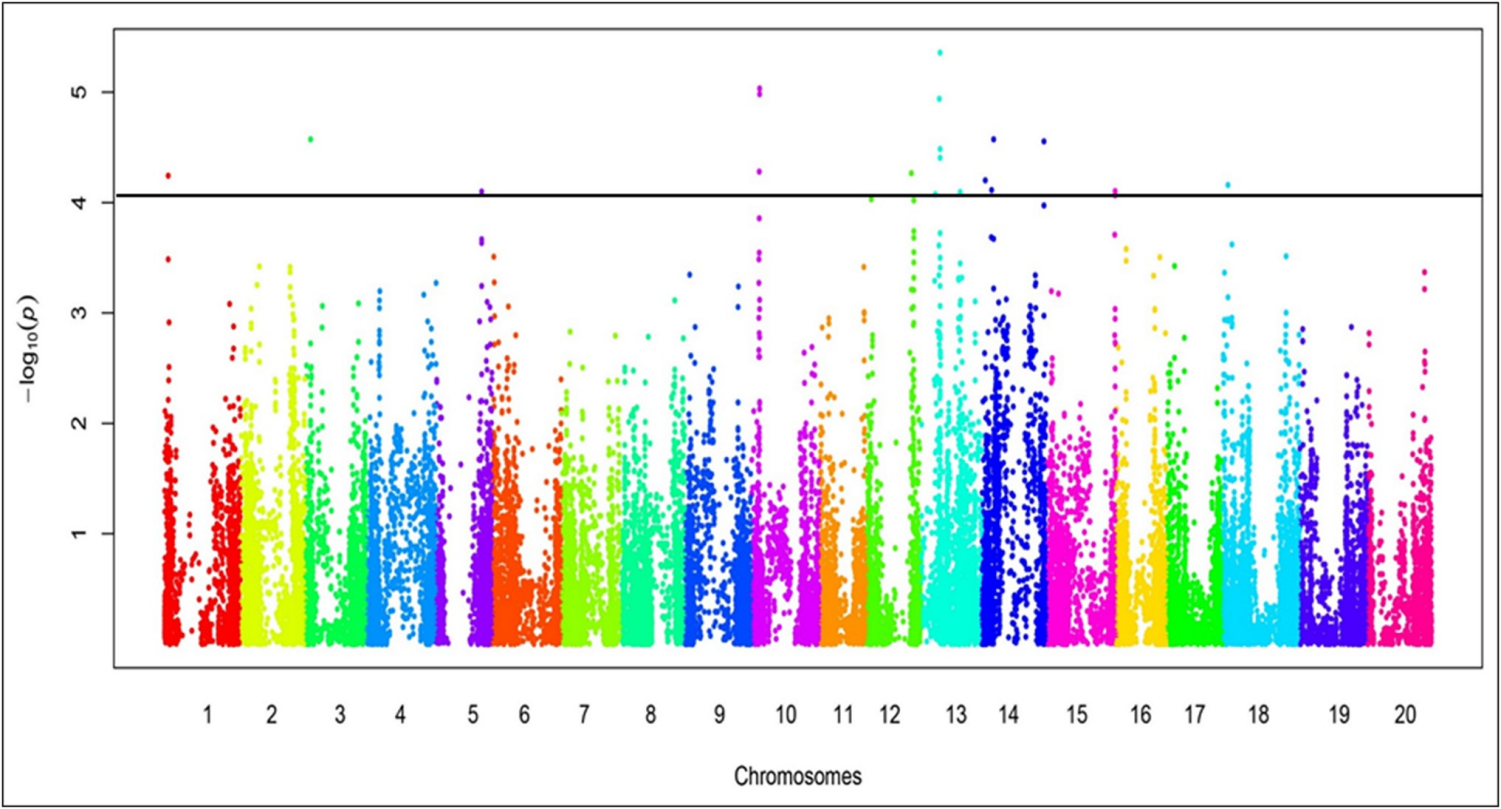
Manhattan plot of the SNPs associated with the foliar SDS scores of the 254 PI lines. The − log10 *p*-values from a genome-wide scan are plotted against the positions of each of the SNPs on 20 chromosomes. The horizontal blue line indicates the genome-wide significance threshold (FDR < 0.05). SNPs in Manhattan plot were placed in kb unit.

**Fig 5:**
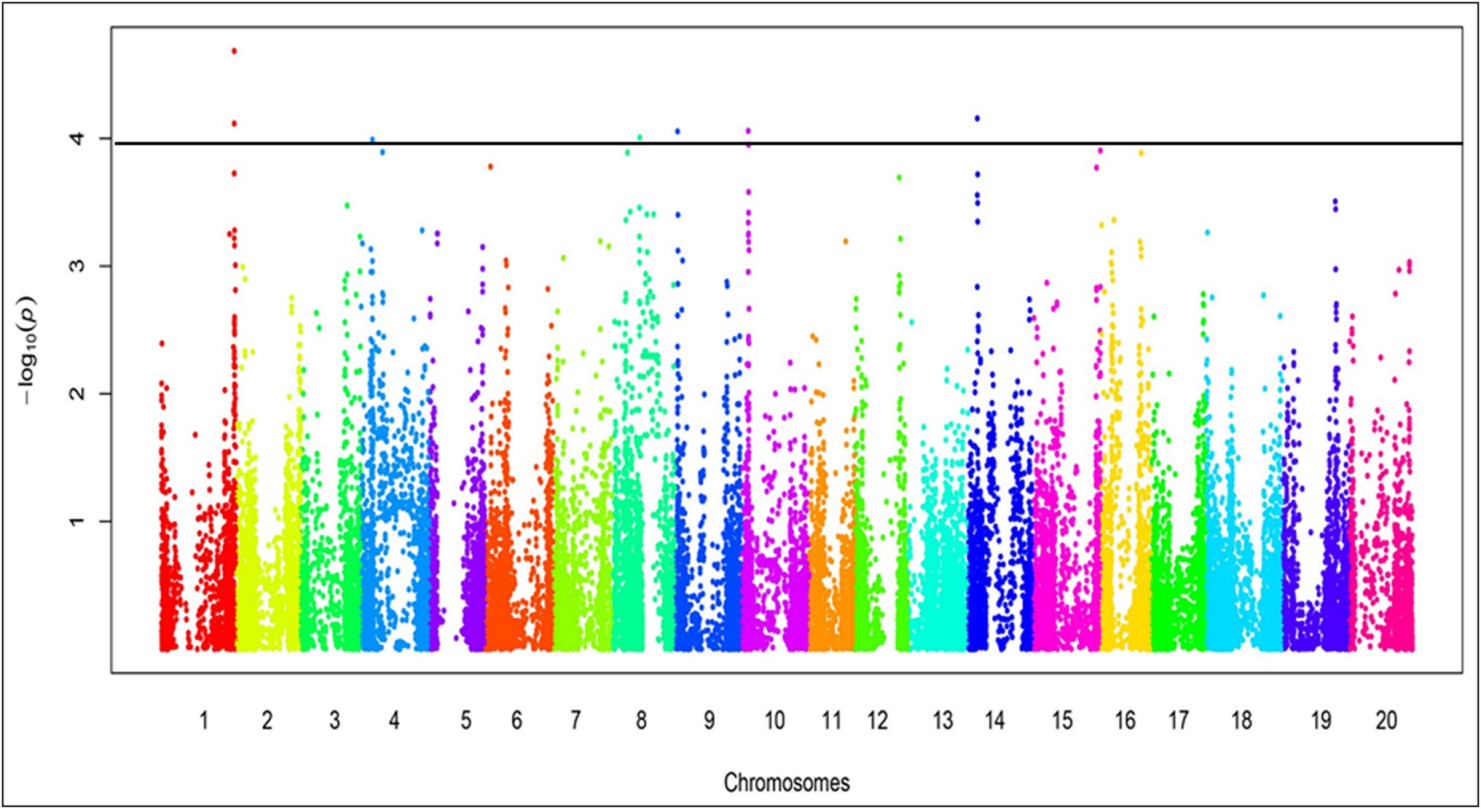
Manhattan plot of the SNPs associated with the root rot severities of 254 PI lines. The − log10 *p*-values from a genome-wide scan are plotted against the positions of each of the SNPs on 20 chromosomes. The horizontal blue line indicates the genome-wide significance threshold (FDR < 0.05). SNPs in Manhattan plot were placed in kb unit.

**Fig 6:**
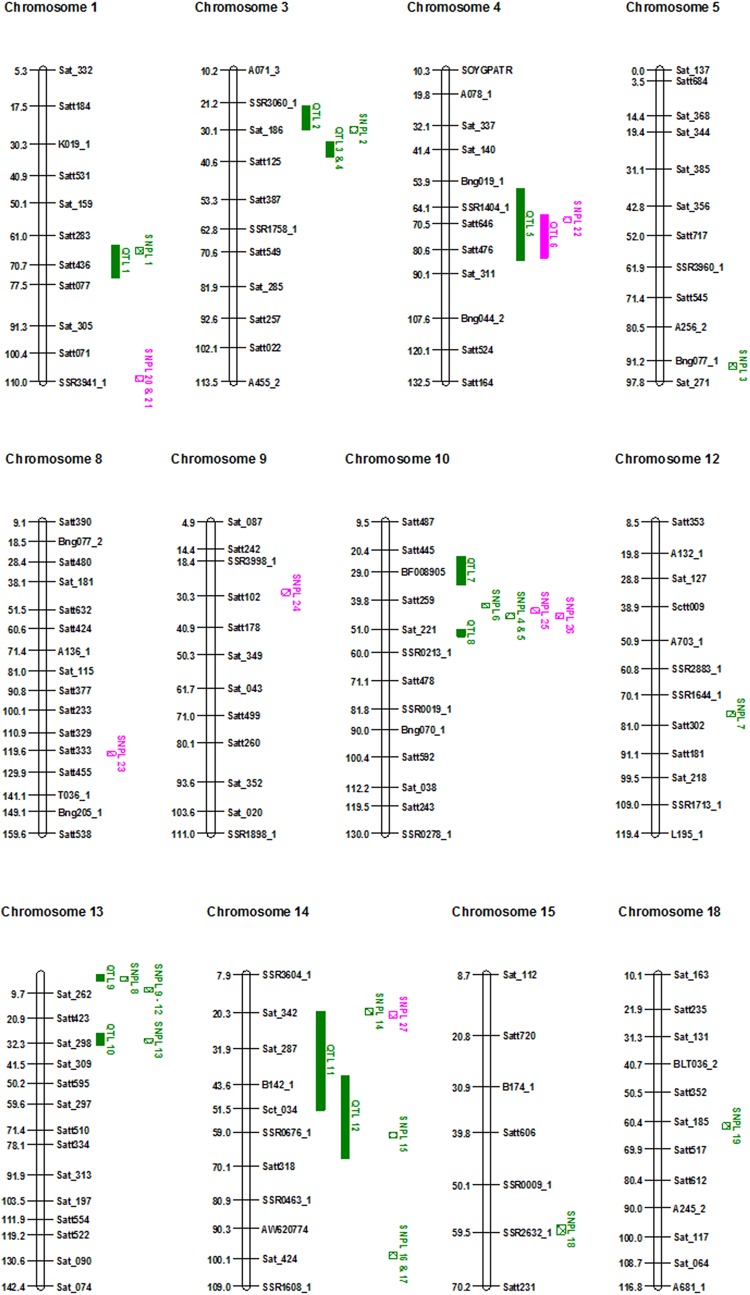
The genetic map of the *F*. *virguliforme* foliar SDS and root rot resistance SNPL. Green checked box, foliar SDS SNPL; Pink checked box, root rot SNPL. SNPL are shown with numerical numbers (Tables 1 and 2). Previously identified foliar SDS and root rot resistance QTL are shown with solid green and pink boxes, respectively (S2 Table). The genetic map is generated in centi-Morgan (cM) unit.

GWAS identified 19 SNP loci (SNPL) that significantly associated with 14 genomic regions encoding foliar SDS resistance (Fig 4, Table 1) and eight SNPL associated with seven genomic regions for root rot resistance (Fig 5, Table 2). Of these 27 SNPL, six SNPL for foliar SDS resistance and two SNPL for root rot resistance co-mapped to previously identified QTL for SDS resistance (Fig 6). This study identified 13 SNPL associated with eight novel genomic regions containing foliar SDS resistance genes and six SNPL with five novel regions for root-rot resistance. Of the 27 SNPL identified, five generated nonsynonymous mutations in five genes: (i) three of which mapped to previously identified QTL for foliar SDS resistance and (ii) two mapped to two novel regions containing root rot resistance genes (Table 3).

**Table 1:** Details of 19 SNPs that are associated with foliar SDS resistance.

SNP #	SNPs^a^	Physical and genetic map position^b^	*p*-value^c^	R^2^ value (%)	SNP in gene^d^	Protein identification	Possible role in plant defense^e^
1	**ss715579740_A_G**	48684677 (65–67)	1.53E-04	5.95	*Glyma*.*01g149600*^*i*^	LRR–receptor protein	Pathogen recognition and activation of plant immunity [56]
2	**ss715585711_A_C**	3850435 (26–28)	1.09E-05	7.52	*Glyma*.03g033200*	Polynucleotidyl transferase/ ribonuclease H-like protein	Anti-fungal activity / pathogen resistance [57]
3	ss715591951_C_T	39907435 (92–94)	8.23E-04	6.30	*Glyma*.*05g219600*^*n*^	Heat Shock Protein 70	Quality control of PRR and R proteins [58]
4	ss715608333_A_G	5808402 (45–47)	5.51E-05	9.13	*Glyma*.*10g061800*^*n*^	Pathogenesis-related thaumatin superfamily protein	Anti-fungal activity / pathogen resistance [59]
5	ss715608329_C_T	5781593 (45–47)	3.57E-05	8.65	*Glyma*.*10g061700*^*n*^	Pathogenesis-related thaumatin superfamily protein	Anti-fungal activity / pathogen resistance [59]
6	ss715608298_G_A	5569766 (41–43)	1.43E-04	6.51	*Glyma*.*10g060100*^*n*^	Glutamine synthetase	Disease defense response [60]
7	ss715612543_C_T	35078375 (76–78)	1.46E-04	7.14	*Glyma*.*12g189100*^*i*^	PPR repeat protein	Disease resistance genes [61]
8	**ss715613738_G_T**	11106359 (2–4)	8.52E-04	8.12	*Glyma*.13g035700**	LRR–receptor protein	Pathogen recognition and activation of plant immunity [56]
9	ss715617107_A_G	14610894 (7–9)	1.45E-05	6.09	*Glyma*.*13g050200*^*i*^	LRR–receptor protein	Pathogen recognition and activation of plant immunity [56]
10	ss715617111_A_C	14577952 (7–9)	1.16E-04	6.33	*Glyma*.*13g049800*^*i*^	AAA+-type ATPase	Stomatal aperture closing during biotic and abiotic stress [62]
11	ss715617189_C_T	14040386 (7–9)	8.06E-05	7.02	*Glyma*.*13g046200*^*n*^	Ribulose-1,5-bisphosphate carboxylase	Surveillance agent of pathogen invasion [63]
12	ss715617218_C_T	13855912 (7–9)	1.08E-04	5.12	*Glyma*.*13g044800*^*n*^	Sterol C5 desaturase	Regulation of the induction of defense responses [64]
13	**ss715615487_C_T**	18567932 (30–32)	7.11E-04	7.97	*Glyma*.13g079100**	LRR–receptor protein	Pathogen recognition and activation of plant immunity [56]
14	**ss715618125_A_G**	2598265 (19–21)	2.19E-04	6.91	*Glyma*.14g035000**	Unknown function	-
15	**ss715617333_C_T**	9890873 (59–61)	1.13E-04	8.64	*Glyma*.*14g100700*^*n*^	LRR–receptor protein	Pathogen recognition and activation of plant immunity [56]
16	ss715619446_ C_T	47828866 (100–102)	1.00E-05	6.06	*Glyma*.14g213300*	Glutamine synthetase	Disease defense response [60]
17	ss715619290_C_T	46682133 (98–100)	7.09E-04	5.50	*Glyma*.*14g201400*^*n*^	Unknown function	-
18	ss715622696_G_T	50888956 (58–60)	8.25E-04	5.61	*Glyma*.*15g271700*^*i*^	Thioredoxin superfamily protein	Homeostasis of apoplastic ROS in response to pathogen attack [65]
19	ss715631294_C_T	47670147 (61–63)	3.92E-04	6.52	*Glyma*.*18g198500*^*i*^	PPR repeat protein	Disease resistance genes [61]

^a^SNP dataset reported by Song et al. 2013 [52] is available at www.soybase.org. SNPs with bold font were mapped to previously reported QTL for SDS resistance.

^b^Physical position (bp) of the SNP on the soybean reference genome Glyma.Wm82.a2 (Gmax2.0) (www.soybase.org). The values in parentheses are cM distances of the genomic intervals containing putative SDS resistance genes.

^c^*p*-values were obtained from the Manhattan plot (Fig 4).

^d^SNP located in intron (^i^), 5’-UTR or 3’-UTR (*); SNP caused non-synonymous mutation at amino acid level (**); the nearest annotated gene within 20 kb of an identified SNP (^n^).

^e^Possible role of the annotated genes in plant defense. Corresponding literatures are listed in the parentheses.

**Table 2:** Details of eight SNPs that are associated with root rot resistance.

SNP #	SNPs^a^	Physical and genetic map position^b^	*p*-value^c^	R^2^ value (%)	SNP in gene^d^	Protein identification	Possible role in plant defense^e^
20	ss715580536_T_C	55158751 (108–110)	9.82E-05	7.37	*Glyma*.01g222700*	Ferric reductase; NADH/NADPH oxidase	Involved in ROS production [66]
21	ss715580538_A_G	55177993 (108–110)	7.95E-04	6.23	*Glyma*.01g222900**	Late embryogenesis abundant (LEA) hydroxyproline-rich glycoprotein family	Stress induced; upregulated in defense response [67]
22	**ss715589511_C_T**	8818094 (68–70)	1.31E-04	6.02	*Glyma*.*04g097400*^*i*^	PPR repeat	Disease resistance genes [61]
23	ss715600599_A_G	21041383 (120–122)	2.02E-04	7.26	*Glyma*.*08g244500*^*i*^	UDP-glucuronosyl & UDP-glucosyl transferase	Disease defense response [68]
24	ss715604938_A_G	48977375 (28–30)	8.40E-04	7.72	*Glyma*.09g052700*	K+ potassium transporter	upregulated in defense response [69]
25	ss715608261_C_T	5424024 (43–45)	4.79E-04	8.15	*Glyma*.10g058700**	Heparan-alpha-glucosaminide N-acetyltransferase	Disease defense response [70]
26	ss715608307_A_G	5588824 (45–47)	1.02E-04	9.06	*Glyma*.*10g060200*^*i*^	Glutamine synthetase	Disease defense response [60]
27	**ss715619816_A_G**	6989713 (20–22)	1.70E-04	8.53	*Glyma*.*14g080600*^***n***^	Apoptosis-promoting RNA-binding protein	In programmed cell death upon pathogen infection [71]

^a^SNP dataset reported by Song et al. 2013 [52] is available at www.soybase.org. SNPs with bold font were mapped to previously reported QTL for SDS resistance.

^b^Physical position (bp) of the SNP on the soybean reference genome Glyma.Wm82.a2 (Gmax2.0) (www.soybase.org). The values in parentheses are cM distances of the genomic intervals containing putative SDS resistance genes.

^c^*p*-values were obtained from the Manhattan plot (Fig 5).

^d^SNP located in intron (^i^), 5’-UTR or 3’-UTR (*); SNP caused non-synonymous mutation at amino acid level (**); the nearest annotated gene within 20 kb of an identified SNP (^n^).

^e^Possible role of the annotated genes in plant defense. Corresponding literatures are listed in the parentheses.

**Table 3:** Five nonsynonymous mutations that are mapped to candidate SDS resistance genes.

Gene	Protein ID	Mutation	R^2^ value (%)^1^
**Foliar SDS**			
*Glyma*.*13g035700*	LRR–receptor protein	Ser322Arg	8.12
*Glyma*.*13g079100*	LRR–receptor protein	Glu261Lys	7.97
*Glyma*.*14g035000*	Unknown function	Pro70Leu	6.91
**Root rot**			
*Glyma*.*01g222900*	LEA hydroxyproline-rich glycoprotein family	Ser14Asn	6.23
*Glyma*.*10g058700*	Heparan-alpha-glucosaminide N-acetyltransferase	His201Arg	8.15

^1^R^2^ value (%) defines the % of variation explained by a SNPL or a gene.

Of the three genes containing nonsynonymous mutations and mapped to genomic regions containing previously reported foliar SDS resistance QTL, two encode LRR-receptor proteins and one encodes an unknown protein. Two nonsynonymous mutations (ss715613738, and ss715615487) resulted in Ser322Arg and Glu261Lys mutations in the two LRR-receptor proteins encoded by the *Glyma*.*13g035700* and *Glyma*.*13g079100* genes, respectively (Tables 1 and 3). The non-synonymous mutations were detected in conserved residues of the LRR- proteins (Table 3, S7 and S8 Figs). The nonsynonymous mutation (ss715618125) in *Glyma*.*14g035000* gene encoding an unknown protein caused the Pro70Leu mutation (Tables 1 and 3).

Of the two nonsynonymous mutations localized to two genes associated with root rot resistance, the nonsynonymous mutation (ss715608261) in *Glyma*.*10g058700* encoding a putative heparan-alpha-glucosaminide N-acetyltransferase enzyme resulted in the His201Arg mutation (Tables 2 and 3). The nonsynonymous mutation (ss715580538) in *Glyma*.*01g222900* gene encoding a LEA hydroxyproline-rich glycoprotein resulted in the Ser14Asn mutation (Tables 2 and 3). In this study we also identified 22 additional SNPL, which were mapped to non-coding regions (intron, 3’ UTR and 5’ UTR) or intergenic regions (Tables 1 and 2).

## Discussion

The genetics of resistance to SDS is highly complex. It was reported that a single dominant gene, *Rfs* controls SDS resistance in the soybean cultivar, Ripley under greenhouse conditions [29]. Subsequent studies revealed that SDS resistance is conditioned by additional QTL [17, 19–30]. Recently, it has been reported that SDS is controlled by over 80 QTL and epistasis gene interactions [31–33].

We evaluated a collection of 254 PI lines, pre-selected as putative foliar SDS resistant lines from a collection of over 6,000 PI lines, for foliar and root responses to a mixture of three *F*. *virguliforme* aggressive isolates under growth-chamber conditions. Earlier, preliminary study conducted in the Hartman Lab demonstrated that most of the 254 lines were highly resistant to *F*. *virguliforme* Mont-1 isolate (S5 Fig). Previous studies had showed that different soybean genotypes had varying levels of resistance to different *F*. *virguliforme* isolates [72, 73]. We therefore mixed three aggressive *F*. *virguliforme* isolates, Mont-1, Scott-F2I11a and Clinton-1B, to identify the most SDS resistant soybean lines.

In our study we identified seven highly foliar SDS resistant PIs with foliar disease scores <1.5 and 23 PIs with highly root rot resistant lines with <10% of the total root volume showing rotting. Only one PI line showed foliar SDS score of <1.5 and <10% root rot (S1 Table; Fig 3B). We however identified 10 lines that showed foliar disease scores < 2 and root rot ≤ 10% (S1 Table), which could be ideal resources for breeding SDS resistant lines.

We phenotyped the 254 lines in growth chambers. Disease phenotypes of individual lines were very consistent. We observed broad sense heritabilities of 0.90 and 0.77 were for responses of leaves and roots to the infection by the three isolate mixture. Furthermore, the leaf and root responses of the 254 lines to the *F*. *virguliforme* isolate mixture showed normal distributions (Figs 1 and 2; S6 Fig). Therefore, the phenotypic disease data were suitable for conducting GWAS to identify genetic loci for SDS resistance.

Over 20,000 PI lines including the 254 lines have been genotyped using the Infinium SoySNP50K BeadChip [52]. We used this published genotypic data with our phenotypic data to conduct the GWAS and identified 27 genetic loci (SNPL) for SDS resistance (Tables 1 and 2). Of the 27 SNPL, 19 govern foliar SDS resistance and eight root rot resistance (Tables 1 and 2). We observed that six of these SNPL for foliar SDS resistance and two SNPL for root rot resistance mapped to genomic regions that already have been reported to contain SDS resistance QTL [17, 22, 30, 32, 74–76] (Tables 1 and 2; Fig 6; S2 Table). This study identified 19 SNPL associated with eight novel genomic regions containing foliar SDS resistance genes and five novel regions for root-rot resistance, which will however require validation through genetic mapping studies.

Five of the 27 SNPL resulted changes in amino acid residues in five proteins (Table 3). We investigated if the nonsynonymous mutations affected any conserved amino acid residues that may be involved in gene functions. Comparison of the target proteins with their respective homologous protein sequences revealed that in each of the five proteins, the mutations affected the conserved amino acid residues that may lead to changes in either protein structure or post-translational modification, which could be important for expression of SDS resistance (Table 3, S7–S11 Figs). In three of the five proteins, the conserved amino acid residues, serine or histidine for phosphorylation, were altered by the mutations. Thus, altered functions for these three proteins could be possible leading to SDS susceptibility. The three proteins are: LRR–receptor protein, a LEA hydroxyproline-rich glycoprotein and a heparan-alpha-glucosaminide N-acetyltransferase. In an LRR-receptor and a novel protein with unknown function, lysine was mutated to glutamate and proline was mutated to leucine, respectively. These mutations can change the structures and functions of the two proteins. We however need to validate these genes for their role in SDS resistance through genetic mapping studies and/or study of the mutants generated for these candidate SDS resistance genes.

In our study, we identified two genomics regions to which more than one SNPL mapped. For example, SNPL ss715608333 and ss715608329 are only 26.8 kb apart and located in a 2 cM interval for SDS resistance. SNPL ss715617107, ss715617111, ss715617189 and ss715617218 are located in a 75.5 kb genomic region, one the average with one SNLP in every 25 kb region. We did not identify any non-significant SNPs in these two SNPL-rich regions indicating linkage disequilibrium in these regions associated with QTL for foliar SDS resistance.

In our study, we used only 31,506 SNPs for a genome of 1,150 Mb DNA carrying over 46,000 genes [77, 78]. If the 31,506 SNPs are randomly distributed, we expect to have only 1 SNP in every 36.5 kb soybean genome sequence. In that range, more than one gene is not unusual in the soybean genome, especially in the gene-rich regions; and therefore, causative allele for SDS resistance may not have been detected by the SNP panel used in this study. Thus, some or all of the five candidate genes for SDS resistance identified in this study may not encode SDS resistance. However, these five genes and other SNPL linked to the 13 novel genomic regions can be considered potential molecular markers for enhancing SDS resistance in soybean because linkage disequilibrium in soybean is relatively large, 90 to 574 kb [79].

In summary, the GWAS conducted on foliar SDS and root rot phenotypes of 254 PI lines collected in growth chambers led to identification of 27 SNPL mapped to 21 genomic regions for SDS resistance. Eight of the 21 genomic regions for SDS resistance were previously reported QTL validating the outcomes of our GWAS. Thirteen novel genomic regions containing putative SDS resistance genes and 10 PI lines with both foliar SDS and root rot resistance identified in this investigation will significantly contribute towards breeding soybean cultivars for SDS resistance. Five candidate genes for SDS resistance identified in this study will facilitate further studies for advancing our knowledge of SDS resistance mechanisms in soybean.

## Supporting information

S1 Table PI lines used in this study. (PDF)

S2 Table QTL containing foliar SDS resistance and root rot resistance genes. (PDF)

S1 Fig SDS scoring scheme. The severity of SDS foliar symptoms were scored with an increment of 0.5. The foliar SDS score of each plant was recorded 4 to 5 weeks after planting.
(PDF)

S2 Fig Root rot scoring scheme. Root rot symptoms were scored in a percentage scale from 0 to 100 with an increment of 5 of the total root area.
(PDF)

S3 Fig Neighbor joining tree. Neighbor joining tree showing the relatedness of 254 soybean PI lines was constructed from 31,506 SNPs using Tassel 5.2.33 program. Subgroups are color coded.
(PDF)

S4 Fig Principal components analysis (PCA). (A), PCA showing the extent of relatedness among 254 PI lines with the countries of their origin. (B) PCA showing the extent of relatedness among 254 PI lines based on their maturity groups. (C), PCA showing the extent of relatedness among 254 PI lines based on their foliar SDS score. (D) PCA showing the extent of relatedness among 254 PI lines generated based on root rot.
(PDF)

S5 Fig Distribution of the foliar SDS disease scores among the 254 PI lines identified from a collection of over 6,000 soybean PI lines. (A), The frequency distribution of the 254 selected lines for foliar SDS scores. (B), The Q-Q plot of foliar SDS scores of the 254 PI lines. This experiment was conduct earlier in a greenhouse located in the University of Illinois, Champaign by the Hartman Lab.
(PDF)

S6 Fig Histogram of the residuals and Q-Q plots of the severities of foliar disease scores or root rots (%) among the 254 selected PI lines. (A), The residual plot of the foliar SDS scores among the 254 PI lines. (B), The Q-Q plot of the foliar SDS scores among 254 PI lines. (C), The residual plot of the root rot (%) among the 254 PI lines. (D), The Q-Q plot of the root rot (%) among the 254 PI lines.
(PDF)

S7 Fig The Ser322Arg mutation in a putative LRR-receptor protein encoded by *Glyma*.*13g035700*.*1* is localized to a conserved amino acid residue (highlighted with yellow). The Glyma.13G035700.1 (XP_003543967.1) protein identified in our study is highlighted in green. The list of highly homologous proteins to Glyma.13G035700.1 are: KHN15520.1 (*Glycine soja*); XP_014622059.1 (*Glycine max*); XP_025980869.1 (*Glycine max*); XP_006596249.2 (*Glycine max*); RDX78236.1 (*Mucuna pruriens*); XP_020225386.1 (*Cajanus cajan*); XP_014495638.2 (*Vigna radiata* var. *radiata*); XP_007162178.1 (*Phaseolus vulgaris*); XP_003543967.1 (Glyma.13G035700.1) (*Glycine max*); KHN09050.1 (*Glycine soja*); XP_014505177.1 (*Vigna radiata* var. *radiata*); XP_014505176.1 (*Vigna radiata* var. *radiata*); BAT82169.1 (*Vigna angularis* var. *angularis*); XP_017428871.1 (*Vigna angularis*); KOM48560.1 (*Vigna angularis*); KOM48561.1 (*Vigna angularis*); BAT82170.1 (*Vigna angularis* var. *angularis*); XP_022637426.1 (*Vigna radiata* var. *radiata*); XP_022637938.1 (*Vigna radiata* var. *radiata*); XP_007161408.1 (*Phaseolus vulgaris*); XP_007161410.1 *Phaseolus vulgaris*); XP_003545672.1 (*Glycine max*); KHN35232.1 (*Glycine soja*); RDX65315.1 (*Mucuna pruriens*); XP_020225379.1 (*Cajanus cajan*).
(PDF)

S8 Fig The Glu261Lys mutation in a putative LRR-receptor protein encoded by *Glyma*.*13g079100*.*1* is localized to a conserved amino acid residue (highlighted with yellow). The Glyma.13G079100.1 (KRH18726.1) protein identified in our study is highlighted in green. The list of highly homologous proteins to Glyma.13G079100.1 are: XP_007161165.1 (*Phaseolus vulgaris*); XP_007161182.1 (*Phaseolus vulgaris*); RDX99908.1 (*Mucuna pruriens*); KRH18016.1 (*Glycine max*); KRH18017.1 (*Glycine max*); NP_001238112.1 (*Glycine max*); KRH18018.1 (*Glycine max*); RDX92217.1 (*Mucuna pruriens*); KRH18726.1 (Glyma.13G079100.1) (*Glycine max*); KRH15824.1 (*Glycine max*); XP_014622088.1 (*Glycine max*); NP_001237957.1 (*Glycine max*); XP_025982075.1 (*Glycine max*); KRH05215.1 (*Glycine max*); XP_006600115.2 (*Glycine max*); KRH05216.1 (*Glycine max*); KRH05221.1 (*Glycine max*); KRH05219.1 (*Glycine max*); XP_006601168.1 (*Glycine max*); XP_014625496.1 (*Glycine max*); KRH05237.1 (*Glycine max*); KRH05235.1 (*Glycine max*); XP_014625601.1 (*Glycine max*); XP_014625602.1 (*Glycine max*).
(PDF)

S9 Fig The Pro70Leu mutation in an unknown protein encoded by *Glyma*.*14g035000* is localized to a conserved amino acid residue (highlighted with yellow). The Glyma.14g035000 (KRH14580.1) protein identified in our study is highlighted in green. The list of highly homologous proteins to Glyma.14g035000 are: XP_007141497.1 (*Phaseolus vulgaris*); KOM46644.1 (*Vigna angularis*); RDX61322.1 (*Mucuna pruriens*); KRH14580.1 (Glyma.14g035000) (*Glycine max*); KRH73546.1 (*Glycine max*); AET00215.1 (*Medicago truncatula*); PNX72299.1 (*Trifolium pratense*); GAU22154.1 (*Trifolium subterraneum*); PKI57765.1 (*Punica granatum*); OWM69137.1 (*Punica granatum*); ONI14172.1 (*Prunus persica*); PQP95689.1 (*Prunus yedoensis* var. *nudiflora*); PON97614.1 (*Trema orientale*); POE81190.1 (*Quercus suber*); OIW04185.1 (*Lupinus angustifolius*); OIW02814.1 (*Lupinus angustifolius*); XP_010651515.1 (*Vitis vinifer*a); XP_007146115.1 (*Phaseolus vulgaris*); KOM26446.1 (*Vigna angularis*); KYP52351.1 (*Cajanus cajan*); RDY02770.1 (*Mucuna pruriens*); KHN28152.1 (*Glycine soja*); KRH50598.1 (*Glycine max*); KHN14693.1 (*Glycine soja*); KRG89674.1 (*Glycine max*).
(PDF)

S10 Fig The Ser14Asn mutation in a late embryogenesis abundant (LEA) hydroxyproline-rich glycoprotein encoded by *Glyma*.*01g222900*.*1* is localized to a conserved amino acid residue (highlighted with yellow). The Glyma.01G222900.1 protein identified in our study is highlighted in green. The list of highly homologous proteins to Glyma.01G222900.1 are: XP_006574309.1 (*Glycine max*); XP_006591523.1 (*Glycine max*).
(PDF)

S11 Fig The His201Arg mutation in an heparan-alpha-glucosaminide N-acetyltransferase encoded by *Glyma*.*10g058700* is localized to a conserved amino acid residue (highlighted with yellow). The Glyma.10G058700.1 (KRH32551.1) protein identified in our study is highlighted in green. The list of highly homologous proteins to Glyma.10G058700.1 are: XP_016651648.1 (*Prunus mume*); XP_018814810.1 (*Juglans regia*); XP_008241451.1 (*Prunus mume*); XP_024022989.1 (*Morus notabilis*); POO01767.1 (*Trema orientale*); PON49032.1 (*Parasponia andersonii*); XP_017251878.1 (*Daucus carota* subsp. *sativus*); PKI57622.1 (*Punica granatum*); XP_010039059.1 (*Eucalyptus grandis*); KRH32551.1 (*Glycine max*); XP_025979781.1 (*Glycine max*); XP_017413005.1 (*Vigna angularis*); XP_014511918.1 (*Vigna radiata* var. *radiata*); XP_006595362.1 (*Glycine max*); RCW19085.1 (*Glycine max*); XP_007144374.1 (*Phaseolus vulgaris*); KHN48245.1 (*Glycine soja*); XP_020239895.1 (*Cajanus cajan*); KYP41914.1 (*Cajanus cajan*); XP_004515935.1 (*Cicer arietinum*); XP_024633997.1 (*Medicago truncatula*); XP_004494902.1 (*Cicer arietinum*); XP_007162785.1 (*Phaseolus vulgaris*); XP_020211654.1 (*Cajanus cajan*); KYP70913.1 (*Cajanus cajan*).
(PDF)
