# Supplementary material for: Genome wide association study identifies novel single nucleotide polymorphic loci and candidate genes involved in soybean sudden death syndrome resistance
Source: PLoS One. 2019 Feb 26;14(2):e0212071. doi: 10.1371/journal.pone.0212071 (PMC6391044; doi:10.1371/journal.pone.0212071)
Supplement: S1 Table — (PDF) [file pone.0212071.s001.pdf]

Supplementary Table 1. PI lines used in this study.

PI lines	MG	Country of origin	Color code NJ tree	Foliar SDS data average*	Root rot data average*
PI253658C	I	China	Pink	1.26 F	5.00 E
PI307863	IX	India	Green	1.87 F	6.00 E
PI424446	V	Korea South	Black	1.91 F	6.00 E
PI323559	VIII	India	Yellow	1.63 F	8.00 E
PI424269B	V	Korea South	Black	1.76 F	8.00 E
PI307882E	IX	India	Yellow	1.66 F	9.00 E
PI592909	0	Russian Federation	Pink	1.81 F	9.00 E
PI189955	0	France	Pink	1.70 F	10.00 E
PI458285	V	Korea South	Red	1.90 F	10.00 E
PI424251B	V	Korea South	Black	1.96 F	10.00 E
PI509109	V	Korea South	Black	1.76 F	12.00 E
PI358316C	0	Japan	Blue	1.70 F	13.00 E
PI561290	V	Taiwan	Blue	1.70 F	13.00 E
PI307889E	IX	India	Green	1.73 F	13.00 E
PI424365	V	Korea South	Black	1.75 F	13.00 E
PI509092	V	Korea South	Black	1.50 F	14.00 E
PI407785	V	Korea South	Black	1.50 F	16.00 D
PI567210	000	Russian Federation	Pink	1.53 F	16.00 D
PI159319	V	South Africa	Pink	1.70 F	16.00 D
PI229358	VII	Japan	Blue	1.93 F	16.00 D
PI507221	III	Japan	Blue	1.58 F	17.00 D
PI612622A	I	Poland	Pink	1.66 F	17.00 D
PI507290	V	Japan	Blue	1.76 F	17.00 D
PI567325A	II	China	Pink	1.77 F	17.00 D
PI424378	VI	Korea South	Black	1.80 F	17.00 D

PI423936	III	Japan	Blue	1.51 F	18.00 D
PI416748	II	Japan	Pink	1.34 F	19.00 D
PI639573	VIII	Burundi	Pink	1.54 F	19.00 D
PI549051A	00	Russian Federation	Pink	1.80 F	19.00 D
PI205090	0	Japan	Blue	1.87 F	19.00 D
PI423742	V	Korea South	Black	1.91 F	19.00 D
PI549074	00	China	Pink	1.38 F	20.00 D
PI340903	IX	Thailand	Green	1.60 F	20.00 D
PI567323B	II	China	Pink	1.68 F	20.00 D
PI592915	00	China	Pink	1.70 F	20.00 D
PI594401C	III	China	Red	1.73 F	20.00 D
PI509091A	V	Korea South	Blue	1.86 F	20.00 D
PI507575	VI	Japan	Blue	1.86 F	20.00 D
PI497953	X	India	Yellow	1.43 F	21.00 D
PI205911	VIII	Thailand	Green	1.59 F	21.00 D
PI506552	V	Japan	Blue	1.70 F	21.00 D
PI290142	0	Hungary	Pink	1.83 F	21.00 D
PI522192B	0	Moldova	Pink	1.88 F	21.00 D
PI417482	III	Japan	Yellow	1.30 F	22.00 D
PI605847	IV	Vietnam	Yellow	1.90 F	22.00 D
PI605839A	IV	Vietnam	Yellow	1.63 F	23.00 C
PI232997	0	Japan	Pink	1.84 F	23.00 C
PI424182B	VI	Korea South	Black	1.90 F	23.00 C
PI393535	III	Japan	Black	1.65 F	24.00 C
PI603712	0	China	Red	1.76 F	24.00 C
PI417464	V	Japan	Blue	1.82 F	24.00 C
PI603424D	I	China	Red	1.90 F	24.00 C
PI438461	00	Romania	Pink	1.50 F	25.00 C

PI561387	V	Japan	Pink	1.73 F	25.00 C
PI458168	V	Korea South	Black	1.76 F	25.00 C
PI594696A	IV	China	Yellow	1.77 F	25.00 C
PI408311-2	V	Korea South	Black	1.98 F	25.00 C
PI423760	V	Korea South	Black	1.48 F	26.00 C
PI506952	VI	China	Blue	1.53 F	26.00 C
PI603753B	III	China	Red	1.74 F	26.00 C
PI518728	V	China	Yellow	1.84 F	26.00 C
PI340898A	IX	Thailand	Green	1.50 F	27.00 C
PI437563	III	China	Red	1.68 F	27.00 C
PI243547	0	Germany	Blue	1.73 F	27.00 C
PI567374	IV	China	Pink	1.76 F	27.00 C
PI307889D	IX	India	Green	1.83 F	27.00 C
PI307856	IX	India	Green	1.90 F	27.00 C
PI507263	VI	Japan	Blue	1.97 F	27.00 C
PI423940	II	Japan	Blue	1.99 F	27.00 C
PI341259	IX	Tanzania	Green	1.92 F	28.00 C
PI340902	IX	Thailand	Green	1.66 F	29.00 C
PI290156	000	Hungary	Blue	1.80 F	30.00 C
PI567171	00	China	Pink	1.77 F	38.00 B
PI567649	III	China	Red	1.36 F	56.00 A
PI408084A	V	Korea South	Black	2.00 F	28.00 C
PI398895	V	Korea South	Black	2.00 F	29.00 C
PI175188	VII	India	Yellow	2.00 F	15.00 D
PI307870B	IX	India	Green	2.00 F	21.00 D
PI507154	V	Japan	Blue	2.01 F	12.00 E
PI423847	V	Korea South	Black	2.02 F	27.00 C
PI417404	V	Japan	Blue	2.02 F	28.00 C

PI408098	V	Korea South	Black	2.03 F	30.00 C
PI593953	I	China	Pink	2.04 F	18.00 D
PI507531	II	Japan	Blue	2.04 F	13.00 E
PI307892	IX	India	Green	2.04 F	28.00 C
PI437886B	II	China	Pink	2.06 F	21.00 D
PI458162	V	Korea South	Black	2.06 F	18.00 D
PI566966B	IX	Indonesia	Green	2.06 F	26.00 C
PI417156	V	Japan	Blue	2.07 F	21.00 D
PI323558	VII	India	Yellow	2.07 F	21.00 D
PI507275	VI	Japan	Blue	2.08 F	24.00 C
PI205901A	IX	Thailand	Green	2.08 F	5.00 E
PI340900A	IX	Thailand	Green	2.09 F	10.00 E
PI509110B	V	Korea South	Black	2.11 E	16.00 D
PI408180-1	V	Korea South	Black	2.12 E	16.00 D
PI071850-1	III	China	Pink	2.16 E	28.00 C
PI408183	V	Korea South	Black	2.16 E	16.00 D
PI605804B	IV	Vietnam	Yellow	2.17 E	31.00 C
PI467320	I	China	Pink	2.20 E	7.00 E
PI458271	V	Korea South	Black	2.20 E	24.00 C
PI507117B	VI	Japan	Blue	2.20 E	21.00 D
PI239484	IX	India	Green	2.20 E	7.00 E
PI495018	IX	China	Green	2.20 E	16.00 D
PI307889A	IX	India	Green	2.20 E	24.00 C
PI407655B	II	China	Pink	2.23 E	10.00 E
PI612742	III	China	Pink	2.23 E	27.00 C
PI205901B	IX	Thailand	Green	2.23 E	28.00 C
PI085625	II	Korea South	Blue	2.26 E	38.00 B
PI567429B	III	China	Red	2.28 E	29.00 C

PI307843B	IX	India	Green	2.29 E	22.00 D
PI181536	I	Japan	Blue	2.30 E	28.00 C
PI424183	V	Korea South	Black	2.32 E	12.00 E
PI398343	V	Korea South	Black	2.32 E	34.00 B
PI423773	VI	Korea South	Black	2.32 E	34.00 B
PI281897B	IX	Indonesia	Green	2.32 E	37.00 B
PI307844	IX	India	Green	2.33 E	10.00 E
PI587954	IX	China	Yellow	2.33 E	27.00 C
PI307878A	IX	India	Green	2.33 E	30.00 C
PI103079	V	China	Red	2.35 E	23.00 C
PI507545	V	Japan	Blue	2.36 E	22.00 D
PI578505	II	China	Pink	2.38 E	9.00 E
PI594696B	IV	China	Yellow	2.40 E	31.00 C
PI408091	V	Korea South	Black	2.40 E	31.00 C
PI281905	IX	Malaysia	Green	2.40 E	18.00 D
PI341258	IX	Tanzania	Green	2.40 E	19.00 D
PI281911C	IX	Philippines	Green	2.40 E	25.00 C
PI215688	IX	Israel	Black	2.40 E	30.00 C
PI307876B	IX	India	Green	2.40 E	32.00 C
PI281899B	IX	Malaysia	Green	2.45 E	26.00 C
PI341261	IX	Tanzania	Green	2.47 E	28.00 C
PI374191	X	India	Yellow	2.47 E	31.00 C
PI518711	II	China	Pink	2.49 E	26.00 C
PI603424A	0	China	Pink	2.50 E	58.00 A
PI612708C	I	China	Pink	2.50 E	26.00 C
PI561377	III	Japan	Pink	2.50 E	28.00 C
PI605842B	IV	Vietnam	Yellow	2.50 E	32.00 C
PI507324	V	Japan	Blue	2.50 E	17.00 D

PI506757	V	Japan	Blue	2.50 E	20.00 D
PI549025	V	China	Black	2.50 E	21.00 D
PI398911	V	Korea South	Black	2.50 E	29.00 C
PI423875	V	Japan	Blue	2.50 E	30.00 C
PI561380	VI	China	Red	2.50 E	26.00 C
PI240662A	IX	Tanzania	Yellow	2.50 E	7.00 E
PI567280	III	Korea South	Black	2.51 E	26.00 C
PI407993	V	Korea South	Black	2.51 E	25.00 C
PI424491B	V	Korea South	Black	2.53 E	13.00 E
PI603706B	IV	China	Red	2.55 E	22.00 D
PI307899B	IX	India	Green	2.56 E	28.00 C
PI341262	IX	Tanzania	Green	2.57 E	12.00 E
PI603746	II	China	Yellow	2.59 E	24.00 C
PI506595A	III	Japan	Blue	2.59 E	24.00 C
PI307882B	IX	India	Yellow	2.60 E	13.00 E
PI307846	IX	India	Green	2.60 E	19.00 D
PI307860	IX	India	Green	2.60 E	31.00 C
PI281901B	IX	Malaysia	Green	2.61 E	13.00 E
PI561229	I	China	Yellow	2.62 E	22.00 D
PI284816B	IX	Malaysia	Green	2.62 E	32.00 C
PI605846A	IV	Vietnam	Yellow	2.63 E	35.00 B
PI507560	V	Japan	Blue	2.63 E	17.00 D
PI587998C	IV	China	Yellow	2.65 E	41.00 B
PI437586C	V	China	Pink	2.67 D	13.00 E
PI307887	IX	India	Green	2.67 D	23.00 C
PI507539	VII	Japan	Blue	2.68 D	21.00 D
PI281899C	IX	Malaysia	Green	2.70 D	17.00 D
PI238109	X	Japan	Blue	2.70 D	16.00 D

PI281897A	IX	Indonesia	Green	2.71 D	22.00 D
PI340904A	IX	Thailand	Green	2.73 D	29.00 C
PI408100A	V	Korea South	Black	2.74 D	38.00 B
PI281901A	IX	Malaysia	Green	2.75 D	27.00 C
PI088823	III	Korea South	Yellow	2.77 D	7.00 E
PI578307C	VII	Nepal	Yellow	2.77 D	15.00 D
PI088777	II	China	Pink	2.79 D	20.00 D
PI307855	IX	India	Green	2.80 D	23.00 C
PI506743	VI	Japan	Blue	2.83 D	20.00 D
PI281911B	IX	Philippines	Green	2.83 D	17.00 D
PI307850B	IX	India	Green	2.83 D	31.00 C
PI417268	II	Japan	Blue	2.86 D	23.00 C
PI507568	VII	Japan	Blue	2.87 D	32.00 C
PI567105	VIII	Indonesia	Green	2.87 D	20.00 D
PI240670	IX	Thailand	Green	2.87 D	16.00 D
PI281913A	IX	Thailand	Green	2.87 D	20.00 D
PI567351B	III	China	Red	2.89 D	27.00 C
PI509099	V	Korea South	Black	2.90 D	21.25 D
PI340000	V	Korea South	Black	2.90 D	27.00 C
PI307842A	IX	India	Green	2.90 D	35.00 B
PI305074	IX	Thailand	Green	2.90 D	38.00 B
PI587716A	IV	China	Red	2.91 D	26.00 C
PI458267	V	Korea South	Black	2.91 D	18.00 D
PI507562	VII	Japan	Blue	2.92 D	18.00 D
PI509102	VI	Korea South	Black	2.93 D	25.00 C
PI561372	V	China	Red	2.97 D	23.00 C
PI281909	IX	Malaysia	Green	2.97 D	26.00 C
PI088310	III	China	Pink	2.99 D	7.00 E

PI307874	IX	India	Green	3.00 D	25.00 C
PI507034	V	Japan	Blue	3.02 D	35.00 B
PI612706B	0	China	Pink	3.03 D	29.00 C
PI279081	VII	South Africa	Pink	3.03 D	21.00 D
PI307847	IX	India	Green	3.07 D	22.00 D
PI458236B	VI	Korea South	Black	3.09 D	26.00 C
PI281891A	IX	Indonesia	Green	3.12 D	24.00 C
PI434976	IX	Nigeria	Green	3.13 D	16.00 D
PI423782	V	Korea South	Black	3.14 D	16.00 D
PI307886	IX	India	Green	3.14 D	41.00 B
PI605846D	IV	Vietnam	Yellow	3.16 D	23.00 C
PI549076A	00	China	Pink	3.17 D	17.00 D
PI594780	IV	China	Yellow	3.17 D	35.00 B
PI587671	VII	China	Red	3.18 D	20.00 D
PI587980A	IV	China	Yellow	3.19 D	37.00 B
PI594802A	IV	China	Pink	3.20 D	20.00 D
PI533605	V	United States	Pink	3.20 D	10.00 E
PI445683	VII	Nepal	Yellow	3.23 D	23.00 C
PI567107A	VIII	Indonesia	Green	3.23 D	22.00 D
PI291291	I	China	Pink	3.24 D	7.00 E
PI281893	X	Indonesia	Green	3.24 D	29.00 C
PI603784	IV	China	Yellow	3.28 D	13.00 E
PI567087A	VIII	Indonesia	Green	3.28 D	22.00 D
PI525454	IV	United States	Pink	3.30 D	24.00 C
PI506805	V	Japan	Blue	3.30 D	17.00 D
PI509101	V	Korea South	Black	3.30 D	20.00 D
PI307837	IX	India	Green	3.33 D	20.00 D
PI471932	VIII	Nepal	Yellow	3.34 D	16.00 D

PI281913B	IX	Thailand	Green	3.34 D	55.00 A
PI468907	I	China	Pink	3.37 D	24.00 C
PI605846C	IV	Vietnam	Yellow	3.37 D	39.00 B
PI082278	III	Korea South	Yellow	3.42 D	5.00 E
PI578464A	IX	Vietnam	Yellow	3.47 C	30.00 C
PI495019	V	China	Red	3.48 C	19.00 D
PI506713	V	Japan	Blue	3.49 C	34.00 B
PI423844	V	Korea South	Black	3.50 C	15.00 D
PI281911A	IX	Philippines	Green	3.50 C	24.00 C
PI192871	VII	Indonesia	Yellow	3.57 C	23.00 C
PI567143	IX	Indonesia	Green	3.57 C	25.00 C
PI567139A	IX	Indonesia	Green	3.58 C	31.00 C
PI417073	V	Japan	Blue	3.63 C	43.00 B
PI227160	V	Korea South	Black	3.66 C	15.00 D
PI507381	VI	Japan	Blue	3.66 C	29.00 C
PI605819A	0	Vietnam	Pink	3.70 C	27.00 C
PI201422	VI	China	Pink	3.70 C	18.00 D
PI567121A	VIII	Indonesia	Green	3.70 C	33.00 C
PI605845B	IV	Vietnam	Yellow	3.71 C	26.00 C
PI594178	I	Japan	Blue	3.77 C	27.00 C
PI506834	V	Japan	Red	3.78 C	17.00 D
PI549023A	V	China	Yellow	3.80 C	15.00 D
PI612708D	I	China	Pink	3.83 C	26.00 C
PI612746	I	China	Yellow	3.87 C	25.00 C
PI594647B	IV	China	Yellow	3.90 C	28.00 C
PI605840G	IV	Vietnam	Yellow	3.96 C	30.00 C
PI603730B	IV	China	Yellow	4.00 C	32.00 C
PI506761	VI	Japan	Blue	4.04 C	20.00 D

PI506621	VI	Japan	Blue	4.08 C	34.00 B
PI416797	V	Japan	Blue	4.28 B	29.00 C
PI229335	V	Japan	Blue	4.43 B	16.00 D
PI424508	V	Korea South	Blue	4.50 B	30.00 C
PI507058	VII	Japan	Blue	5.25 A	21.00 D

*Level of significance of the phenotypic data were caluculated by Scott-Knott test ($p < 0.05$).

*Lines with blue font shows the PI lines with foliar SDS scores <2 with 10% or less root rot.
