# Supplementary material for: Genome wide association study identifies novel single nucleotide polymorphic loci and candidate genes involved in soybean sudden death syndrome resistance
Source: PLoS One. 2019 Feb 26;14(2):e0212071. doi: 10.1371/journal.pone.0212071 (PMC6391044; doi:10.1371/journal.pone.0212071)
Supplement: S2 Table — (PDF) [file pone.0212071.s002.pdf]

Supplementary Table 2. QTL containing foliar SDS resistance and root rot resistance genes.

QTL ID*	Number assigned**	Chromosome	LG	Start cM	End cM	Foliar SDS / Root rot	Population [References]***
SDS 14-1	QTL 1	1	D1a	65.28	75.29	Foliar SDS	MD96-5722 x Spencer [1]
Not in SoyBase	QTL 2	3	N	22.67	29.28	Foliar SDS	Ripley x Spencer [2]
SDS 14-2	QTL 3	3	N	34.50	38.67	Foliar SDS	MD96-5722 x Spencer [1]
SDS 14-3	QTL 4	3	N	34.63	38.67	Foliar SDS	MD96-5722 x Spencer [1]
SDS 13-15	QTL 5	4	C1	57.30	85.37	Foliar SDS	PI438489B x Hamilton [3]; Ripley x Spencer [4]; MN1606 x Spencer [5]
Not in SoyBase	QTL 6	4	C1	67.02	84.80	Root rot	PI438489B x Hamilton [3]
SDS 14-8	QTL 7	10	O	23.36	33.69	Foliar SDS	MD96-5722 x Spencer [1]
SDS 16-7	QTL 8	10	O	51.00	53.66	Foliar SDS	A95-684043 x LS98-0582 [6]
SDS 14-9	QTL 9	13	F	1.00	4.11	Foliar SDS	MD96-5722 x Spencer [1]
Not in SoyBase	QTL 10	13	F	27.87	33.18	Foliar SDS	Essex x Forrest [7]; A95-684043 x LS98-0582 [8]
SDS 14-10	QTL 11	14	B2	19.87	52.03	Foliar SDS	MD96-5722 x Spencer [1]
Not in SoyBase	QTL 12	14	B2	40.60	67.73	Foliar SDS	MN1606 x Spencer [5]

* Previously reported QTL based on the composite genetic map of soybean (SoyBase; www.soybase.org). QTL ID, as assigned in SoyBase.

** Assigned ID for the resistance QTL and SNP (only for this article)

References ***

1. Anderson, J., M. Akond, M.A. Kassem, K. Meksem, and S.K. Kantartzi (2015) Quantitative trait loci underlying resistance to sudden death syndrome (SDS) in MD96-5722 by 'Spencer' recombinant inbred line population of soybean. 3 Biotech 5:203-210 doi:10.1007/s13205-014-0211-3
2. Hashmi, R.Y. (2004) Inheritance of resistance to soybean sudden death syndrome. SDS. in 'Ripley' × 'Spencer' F5 derived lines. PhD Dissertation, Plant Biology, SIUC, Carbondale, USA

3. Kassem, M.A., L. Ramos, L.F. Leandro, G.Y.C. Mbofung, D.L. Hyten, S.K. Kantartzi, R.L. Grier IV, V.N. Njiti, S. Cianzio, and K. Meksem (2012) The 'PI 438489B' by 'Hamilton' SNP-based genetic linkage map of soybean [Glycine max (L.) Merr.] identified quantitative trait loci that underlie seedling SDS resistance. J Plant Genome Sci 1:18–30
4. de Farias Neto, A.L., R. Hashmi, M. Schmidt, S. Carlson, G.L. Hartman, S. Li, R.L. Nelson, and B.W. Diers (2007) Mapping and confirmation of a new sudden death syndrome resistance QTL on linkage group D2 from the soybean genotypes PI567374 and 'Ripley'. Mol Breed 20:53–62
5. Luckew, A.S., S. Swaminathan, L.F. Leandro, J.H. Orf and S.R. Cianzio (2017) 'MN1606SP' by 'Spencer' filial soybean population reveals novel quantitative trait loci and interactions among loci conditioning SDS resistance. Theor Appl Genet 130:2139-2149
6. Swaminathan, S., N.S. Abeysekara, M. Liu, S.R. Cianzio, and M.K. Bhattacharyya (2015) Quantitative trait loci underlying host responses of soybean to Fusarium virguliforme toxins that cause foliar sudden death syndrome. Theor Appl Genet 129(3):495-506 doi: 10.1007/s00122-015-2643-5
7. Yuan J., B.R. Rabia, G. Salas, H. Sharma, A. Srour, and D.A. Lightfoot (2012) New approaches to selecting resistance or tolerance to SDS and Fusarium root rot. J Plant Genome Sci 1:10–17
8. Swaminathan, S., N.S. Abeysekara, J.M. Knight, M. Liu, J. Dong, M.E. Hudson, M.K. Bhattacharyya, S.R. Cianzio (2018) Mapping of new quantitative trait loci for sudden death syndrome and soybean cyst nematode resistance in two soybean populations. Theor Appl Genet 131:1047-1062
