# Supplementary material for: Genome wide association study identifies novel single nucleotide polymorphic loci and candidate genes involved in soybean sudden death syndrome resistance
Source: PLoS One. 2019 Feb 26;14(2):e0212071. doi: 10.1371/journal.pone.0212071 (PMC6391044; doi:10.1371/journal.pone.0212071)
Supplement: S1 Fig — The severity of SDS foliar symptoms were scored with an increment of 0.5. The foliar SDS score of each plant was recorded 4 to 5 weeks after planting. (PDF) [file pone.0212071.s003.pdf]

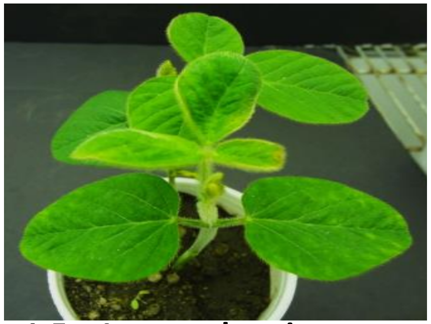

1.5 = Leaves showing very few chlorotic specks

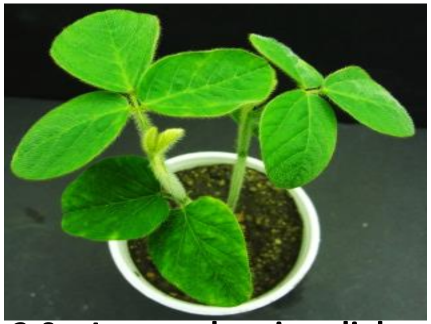

2.0 = Leaves showing slight yellowing/ chlorotic blotches

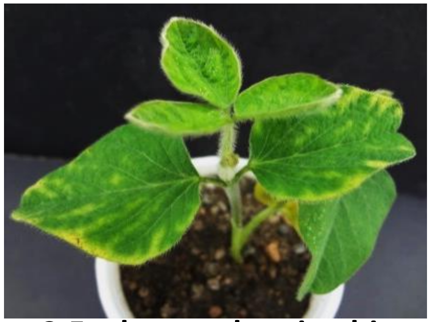

2.5 = leaves showing big chlorotic blotches

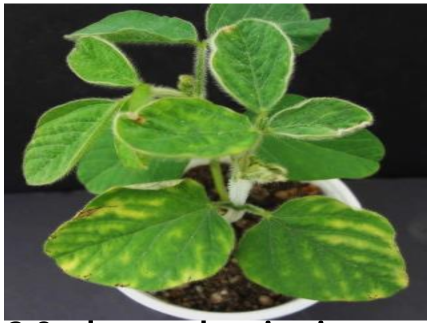

3.0 = leaves showing inter-veinal chlorosis

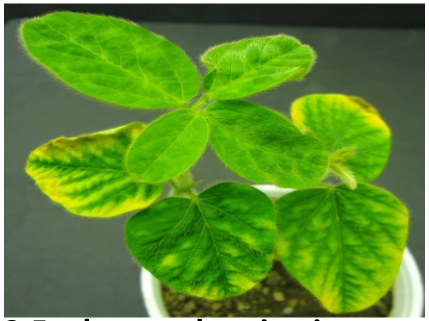

3.5 = leaves showing inter-veinal chlorosis all over

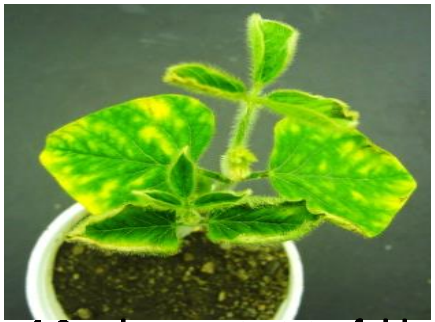

4.0 = leaves start to fold with slight necrosis

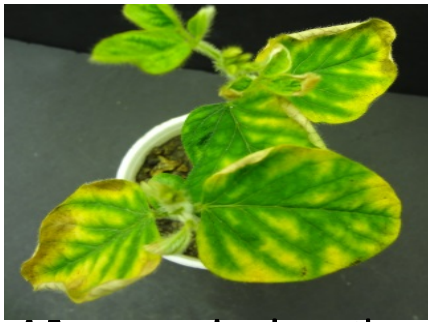

4.5 = necrosis along the portion of leaves

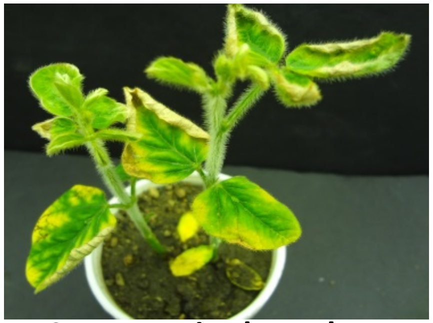

5.0 = necrosis along the entire margin of leaves

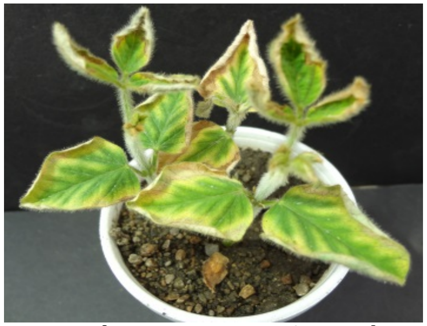

5.5 = heavy necrosis and heavy cupping of leaves

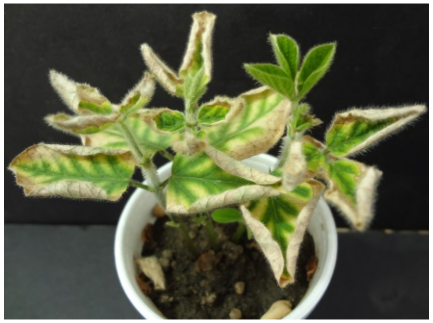

6.0 = heavy necrosis, cupping & rolling of leaves

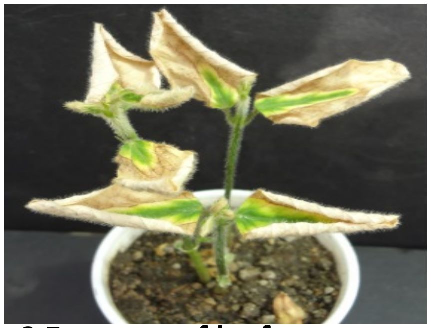

6.5 = most of leaf area necrotic and drying

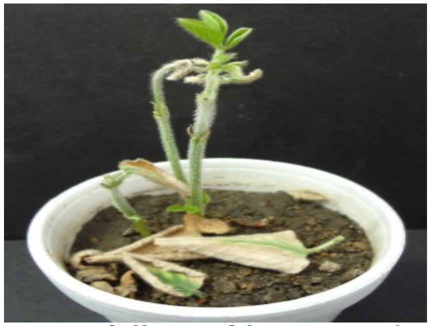

7.0 = falling of leaves and defoliated plants
