# Supplementary figures and images for: Genome wide association study identifies novel single nucleotide polymorphic loci and candidate genes involved in soybean sudden death syndrome resistance
Source: PLoS One. 2019 Feb 26;14(2):e0212071. doi: 10.1371/journal.pone.0212071 (PMC6391044; doi:10.1371/journal.pone.0212071)

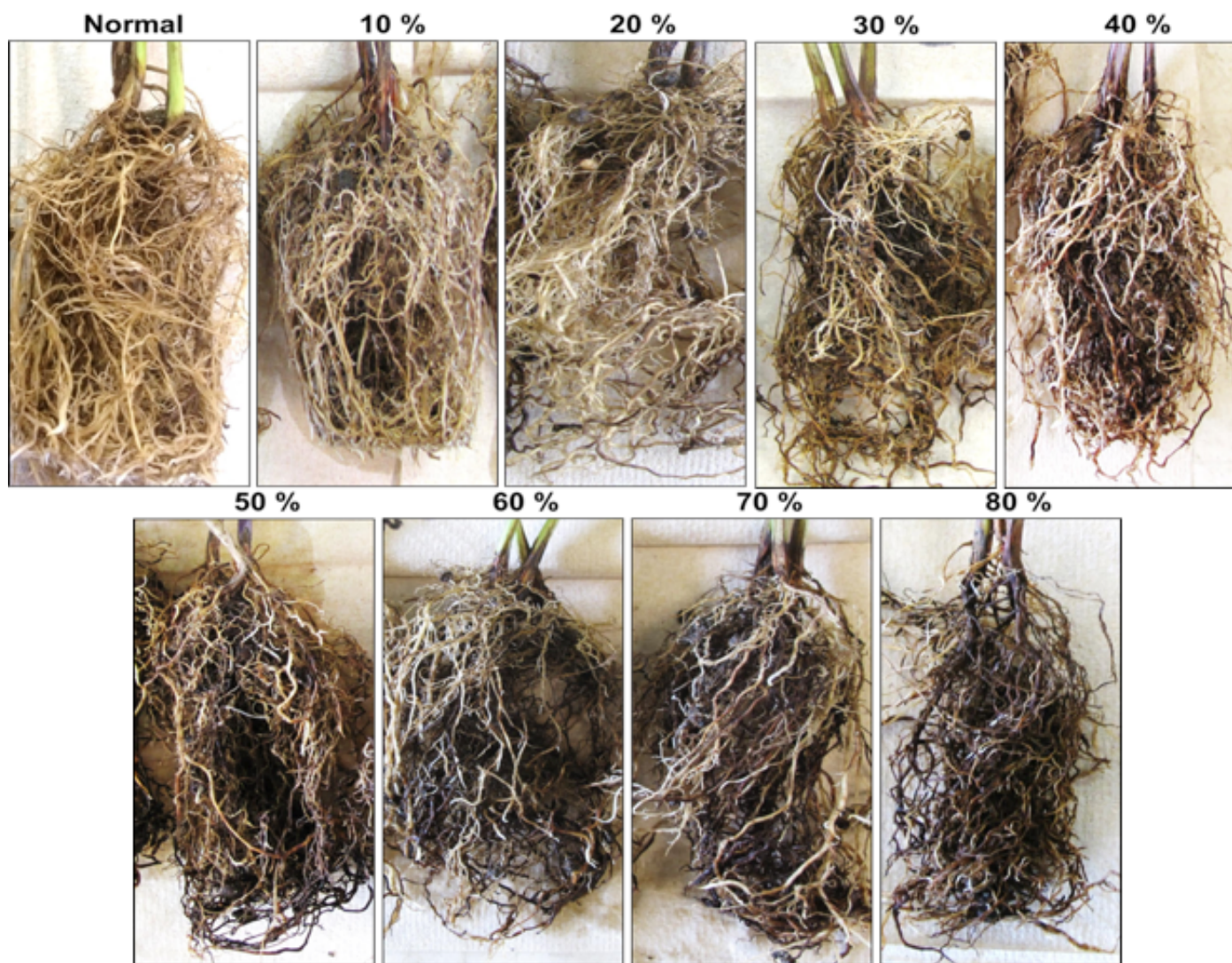

Supplement: S2 Fig — Root rot symptoms were scored in a percentage scale from 0 to 100 with an increment of 5 of the total root area. (PDF) [file pone.0212071.s004.pdf]

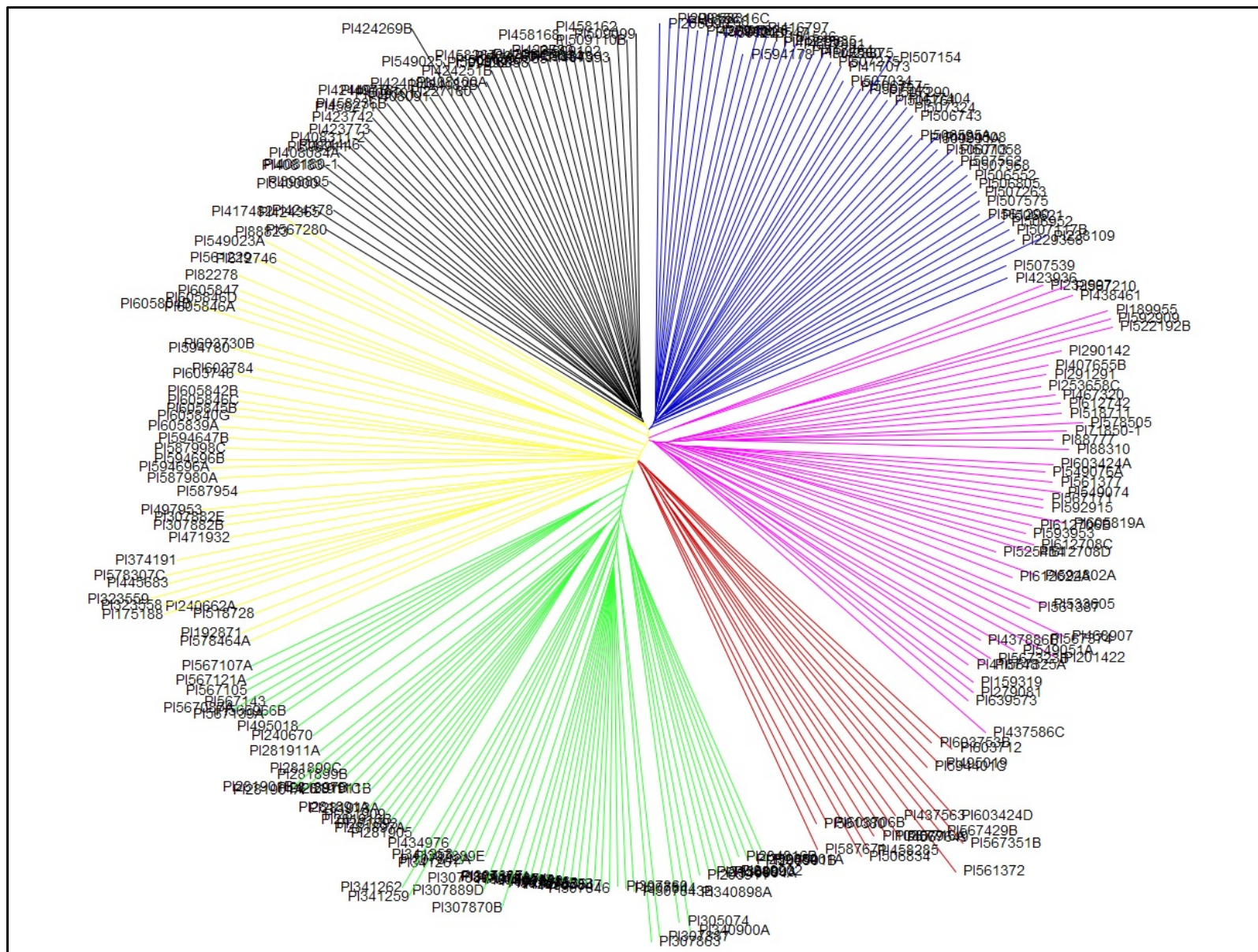

Supplement: S3 Fig — Neighbor joining tree showing the relatedness of 254 soybean PI lines was constructed from 31,506 SNPs using Tassel 5.2.33 program. Subgroups are color coded. (PDF) [file pone.0212071.s005.pdf]

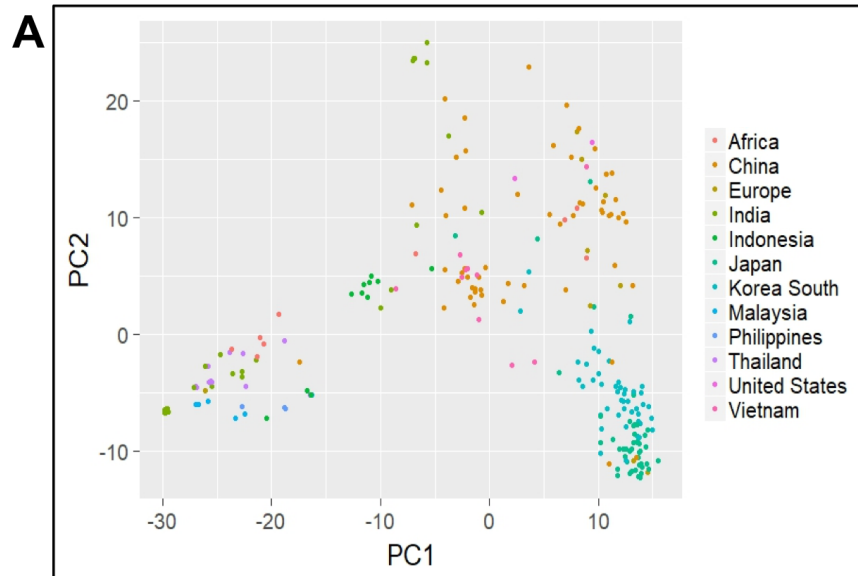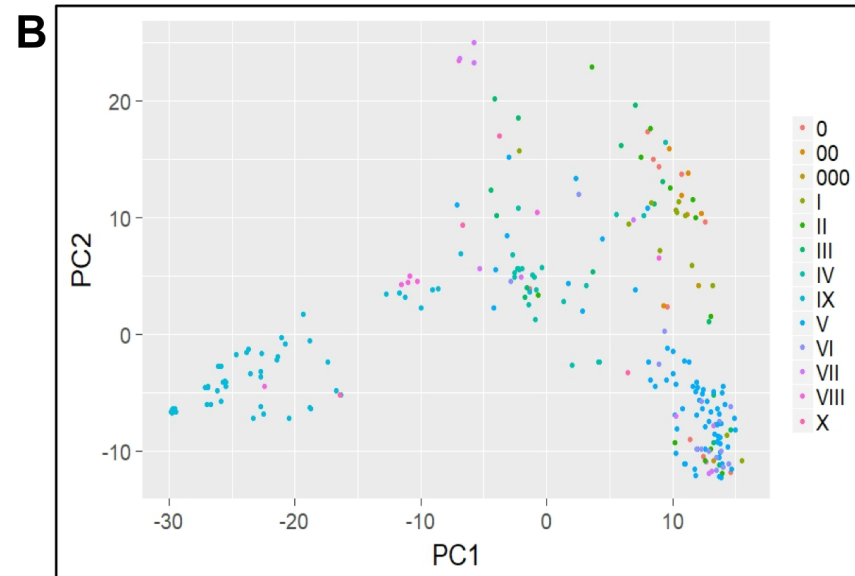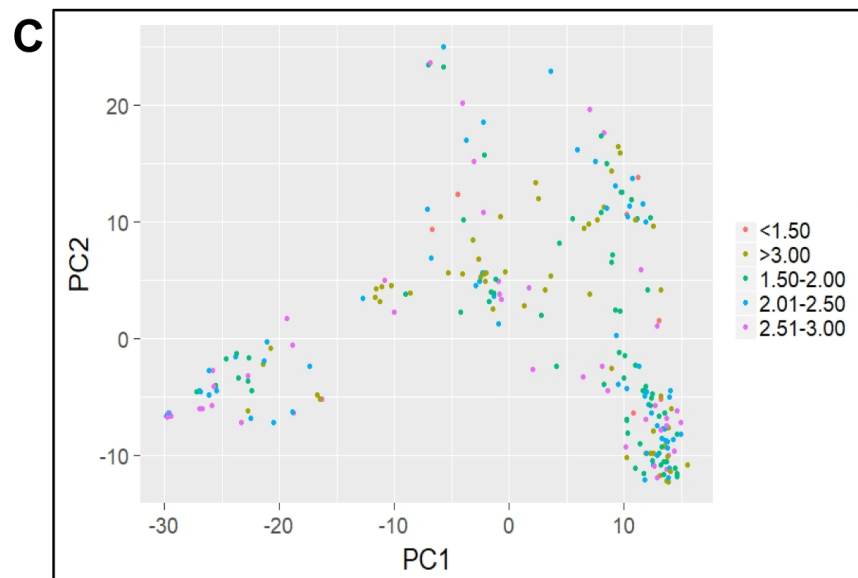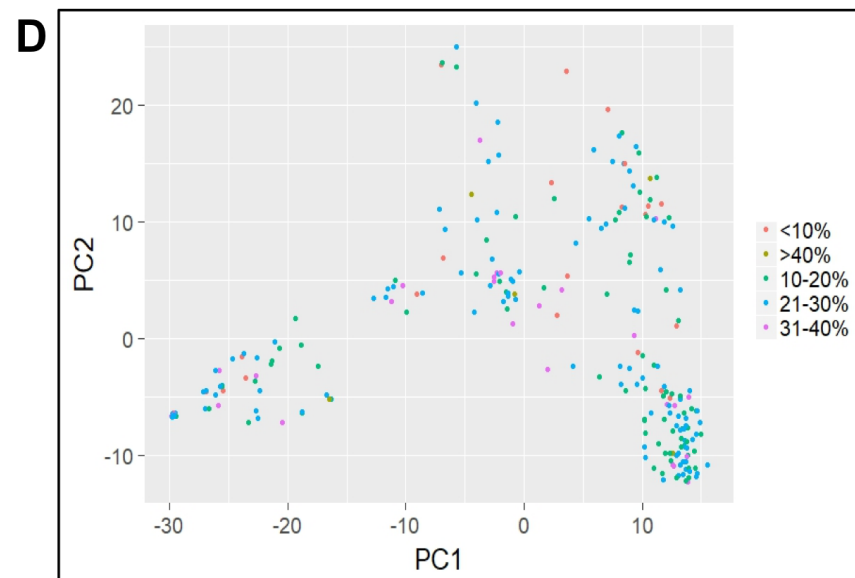

Supplement: S4 Fig — (A), PCA showing the extent of relatedness among 254 PI lines with the countries of their origin. (B) PCA showing the extent of relatedness among 254 PI lines based on their maturity groups. (C), PCA showing the extent of relatedness among 254 PI lines based on their foliar SDS score. (D) PCA showing the extent of relatedness among 254 PI lines generated based on root rot. (PDF) [file pone.0212071.s006.pdf]

**A**

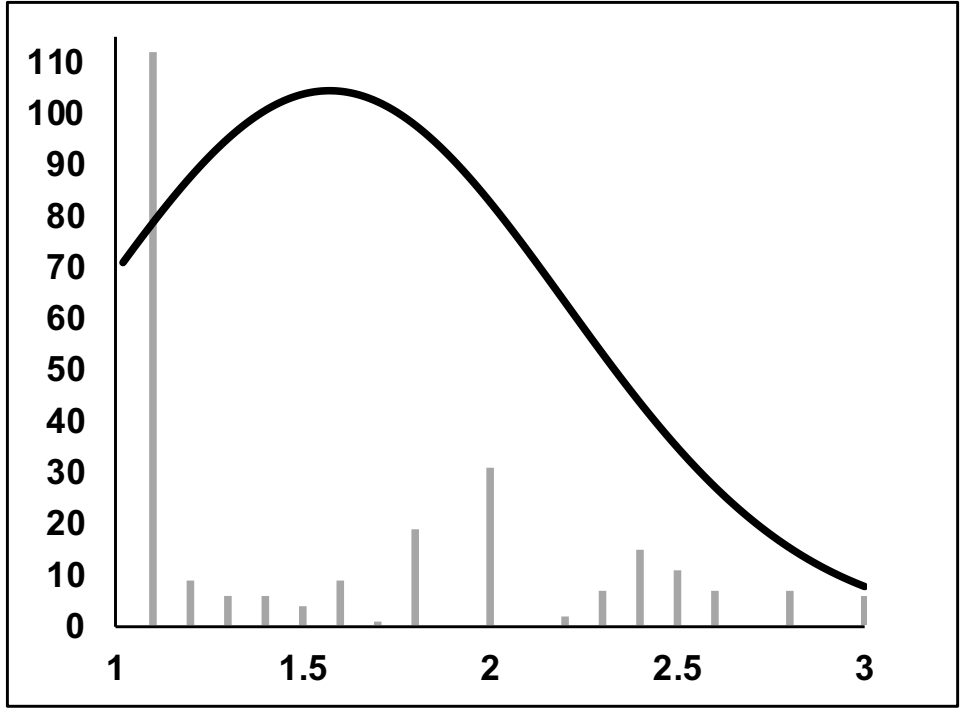

**B**

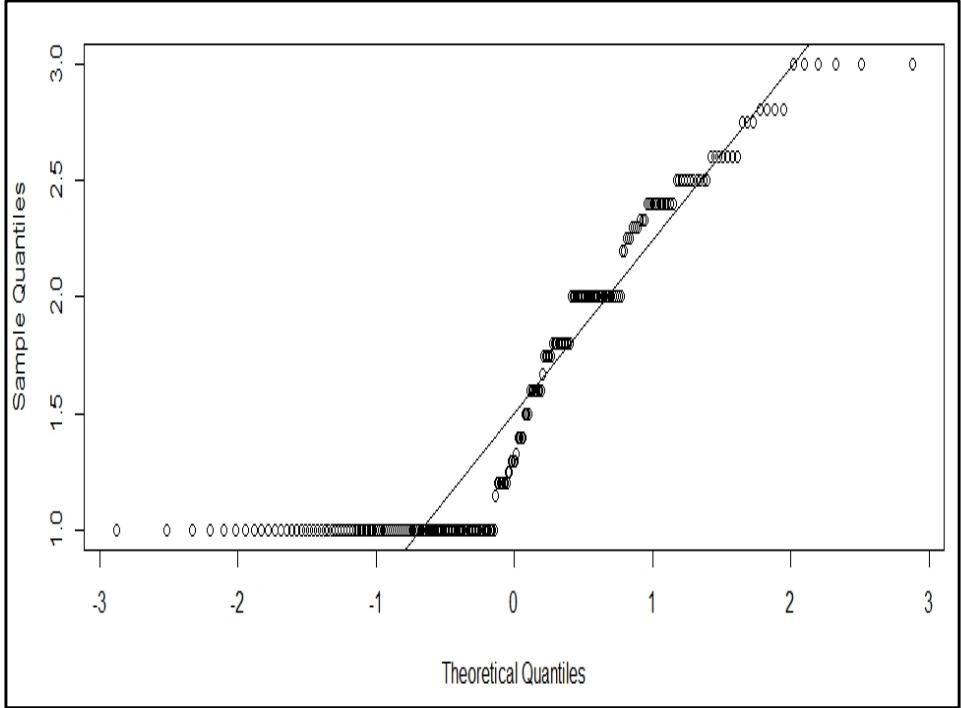

Supplement: S5 Fig — (A), The frequency distribution of the 254 selected lines for foliar SDS scores. (B), The Q-Q plot of foliar SDS scores of the 254 PI lines. This experiment was conduct earlier in a greenhouse located in the University of Illinois, Champaign by the Hartman Lab. (PDF) [file pone.0212071.s007.pdf]

**A**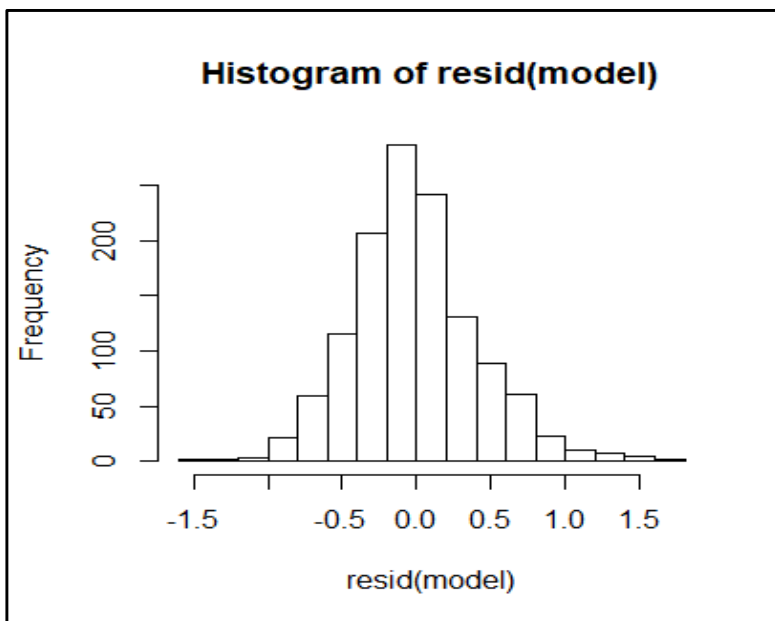**B**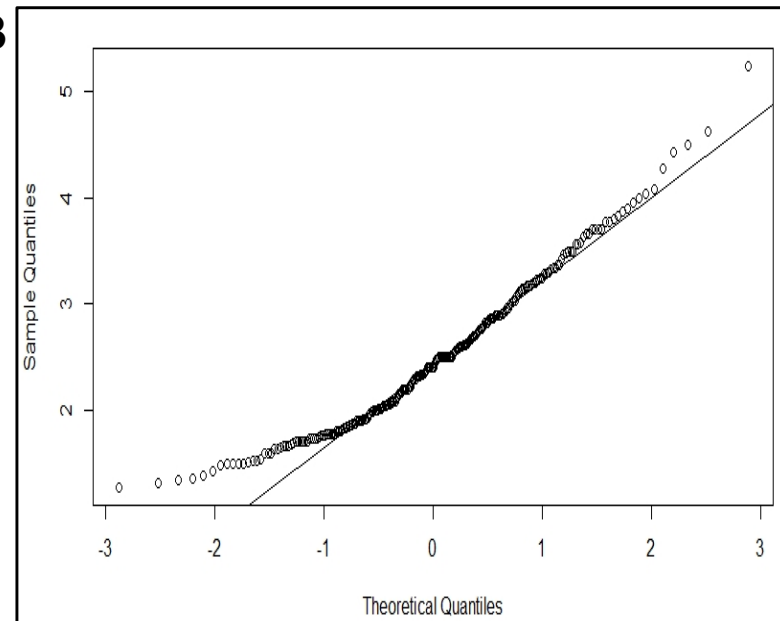**C**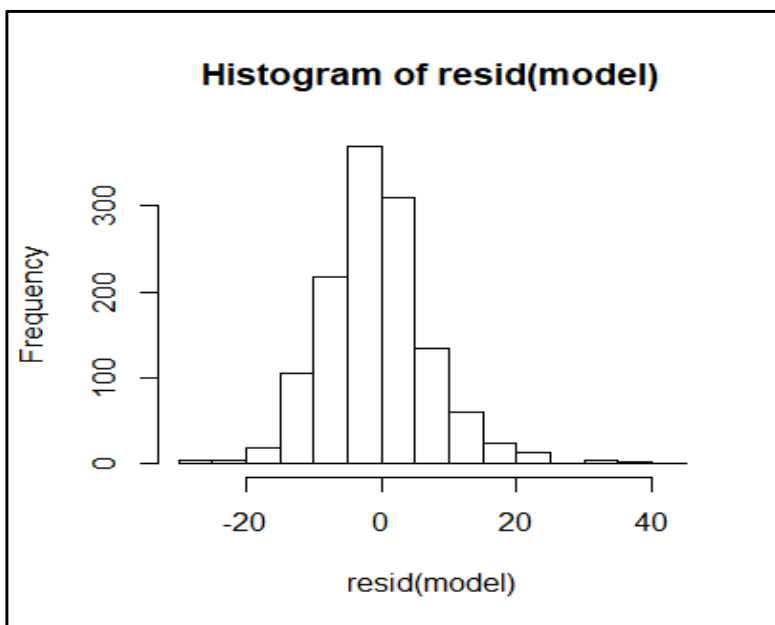**D**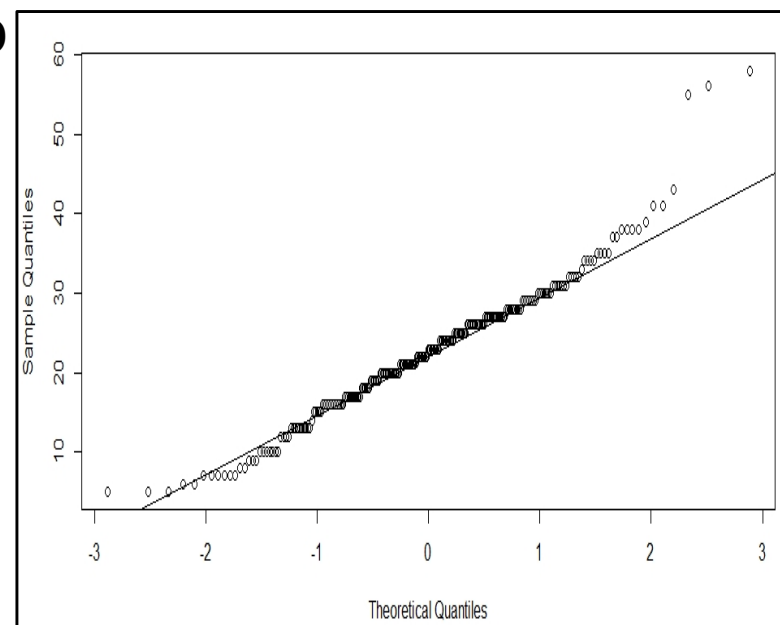

Supplement: S6 Fig — (A), The residual plot of the foliar SDS scores among the 254 PI lines. (B), The Q-Q plot of the foliar SDS scores among 254 PI lines. (C), The residual plot of the root rot (%) among the 254 PI lines. (D), The Q-Q plot of the root rot (%) among the 254 PI lines. (PDF) [file pone.0212071.s008.pdf]
