# Supplementary material for: Genome wide association study identifies novel single nucleotide polymorphic loci and candidate genes involved in soybean sudden death syndrome resistance
Source: PLoS One. 2019 Feb 26;14(2):e0212071. doi: 10.1371/journal.pone.0212071 (PMC6391044; doi:10.1371/journal.pone.0212071)
Supplement: S7 Fig — The Glyma.13G035700.1 (XP_003543967.1) protein identified in our study is highlighted in green. The list of highly homologous proteins to Glyma.13G035700.1 are: KHN15520.1 (Glycine soja); XP_014622059.1 (Glycine max); XP_025980869.1 (Glycine max); XP_006596249.2 (Glycine max); RDX78236.1 (Mucuna pruriens); XP_020225386.1 (Cajanus cajan); XP_014495638.2 (Vigna radiata var. radiata); XP_007162178.1 (Phaseolus vulgaris); XP_003543967.1 (Glyma.13G035700.1) (Glycine max); KHN09050.1 (Glycine soja); XP_014505177.1 (Vigna radiata var. radiata); XP_014505176.1 (Vigna radiata var. radiata); BAT82169.1 (Vigna angularis var. angularis); XP_017428871.1 (Vigna angularis); KOM48560.1 (Vigna angularis); KOM48561.1 (Vigna angularis); BAT82170.1 (Vigna angularis var. angularis); XP_022637426.1 (Vigna radiata var. radiata); XP_022637938.1 (Vigna radiata var. radiata); XP_007161408.1 (Phaseolus vulgaris); XP_007161410.1 Phaseolus vulgaris); XP_003545672.1 (Glycine max); KHN35232.1 (Glycine soja); RDX65315.1 (Mucuna pruriens); XP_020225379.1 (Cajanus cajan). (PDF) [file pone.0212071.s009.pdf]

```
KHN15520.1          CDDKD--SGYGYRCRCNHGYHGNPYLPDGCTDIDECKTANHTCISNKNCRNTIGNYTCFC     318
XP_014622059.1      CDDKD--SDYGYRCMCRDGYEGNPYL--GCIDIDECKTGHHTCVSEKYCLNTNGSHKCFC     322
XP_025980869.1      CDDKD--TDFGYRCRCKDGYEGNPYL--GCTDIDECKTDNHTCISEQNCVNTIGSHTCFC     317
XP_006596249.2      CDDTDSDIDYGYRCRCKDGYEGNPYL--GCTDIDECKTGNHTCISEKNCLNSNGSHRCFC     317
RDX78236.1          CDDKD--SDYGYRCRCNHGYEGNPYL--RCTDIDECKTNNHTCISEQNCLNTDGSHTCFC     310
XP_020225386.1      CNDTD--TDFGYRCTCDDGYEGNPYL--GCTDIDECKTDNHTCISEQNCHNTEGSHICFC     293
XP_014495638.2      CEDPPSAGGNGYLCKCKPGFEGNAYLPQGCTDFPDCKRDKHNCARAEYCHETPGSFECLC     330
XP_007162178.1      CEDPPYANRNGYQCKCKQGFVGNPYLRTGCTDFLECTTGNHNCARDEYCHDIPGSFECLC     327
XP_003543967.1      CDDGDT--DYGYRCRCKDGYEGNSYL--GCTEILECTTRRHNCSREDYCREVRGSFECFC     337
KHN09050.1          CDDGDT--DYGYRCRCKDGYEGNSYL--GCTEILECTTRRHNCSREDYCREVRGSFECFC     319
XP_014505177.1      CEKSPS--EYGYRCKCKKGFQGNAYLPHGCQDILECSSNKHNCDSEDHCRETPGSFECFC     331
XP_014505176.1      CEKSPS--EYGYRCKCKKGFQGNAYLPHGCQDILECSSNKHNCDSEDHCRETPGSFECFC     334
BAT82169.1          CENSPF--EYGYRCKCKQGFQGNAYLLHGCEDIPECTNNKHNCDSEYHCRETLGSFECFC     327
XP_017428871.1      CENSPF--EYGYRCKCKQGFQGNAYLLHGCEDIPECTNNKHNCDSEYHCRETLGSFECFC     324
KOM48560.1          CENSPF--EYGYRCKCKQGFQGNAYLLHGCEDIPECTNNKHNCDSEYHCRETLGSFECFC     327
KOM48561.1          CENSPF--AYGYRCKCKPGFQGNAYLLDGCEDIPECSSNKHNCDSEDHCRETPGSFECFC     319
BAT82170.1          CENSPF--AYGYRCKCKPGFQGNAYLLDGCEDIPECSSNTHNCDSEDHCRETPGSFECFC     327
XP_022637426.1      CENSPF--DYGYRCKCKPGFQGNAYLLDGCQDFHECSNNKHNCDSEDHCRETLGSFECFC     319
XP_022637938.1      CENSPF--EYGYRCKCKPGFQGNAYLLDGCQDIPECSSNKHNCDSEDHCRETPGSFECFC     322
XP_007161408.1      CENSPN--EYGYRCKCNPGFEGNPYLLHGCQDFPECTRNKHNCDSEDHCIETSGSFECFC     318
XP_007161410.1      CDDSPY--EYGYRCKCNPGFDGNAYLLHGCQDIPECARNQHNCDSEDHCIETSGSFECFC     321
XP_003545672.1      CEDSAI--RKSYRCKCKEGYEGNPYHPDGCQDILECTNGRNNCARDEYCRETLGSFQCFC     320
KHN35232.1          CEDSAI--RKSYRCKCKEGYEGNPYHPDGCQDILECTNGRNNCARDEYCRETLGSFQCFC     315
RDX65315.1          CENSAT--GYGYRCKCKAGYEGNPYHPDGCRDILECTTHRHNCASDDYCHEIEGSFQCLC     319
XP_020225379.1      CENSNT--GYGYRCKCKAGFEGNPYLPHGCRDIEECKSGKHNCISEQNCRETEGSFECFC     327
                    *:.       .* * *  *: ** *    * :: :*    :.*     * :  *.. *:*
```
