# Supplementary material for: Genome wide association study identifies novel single nucleotide polymorphic loci and candidate genes involved in soybean sudden death syndrome resistance
Source: PLoS One. 2019 Feb 26;14(2):e0212071. doi: 10.1371/journal.pone.0212071 (PMC6391044; doi:10.1371/journal.pone.0212071)
Supplement: S8 Fig — The Glyma.13G079100.1 (KRH18726.1) protein identified in our study is highlighted in green. The list of highly homologous proteins to Glyma.13G079100.1 are: XP_007161165.1 (Phaseolus vulgaris); XP_007161182.1 (Phaseolus vulgaris); RDX99908.1 (Mucuna pruriens); KRH18016.1 (Glycine max); KRH18017.1 (Glycine max); NP_001238112.1 (Glycine max); KRH18018.1 (Glycine max); RDX92217.1 (Mucuna pruriens); KRH18726.1 (Glyma.13G079100.1) (Glycine max); KRH15824.1 (Glycine max); XP_014622088.1 (Glycine max); NP_001237957.1 (Glycine max); XP_025982075.1 (Glycine max); KRH05215.1 (Glycine max); XP_006600115.2 (Glycine max); KRH05216.1 (Glycine max); KRH05221.1 (Glycine max); KRH05219.1 (Glycine max); XP_006601168.1 (Glycine max); XP_014625496.1 (Glycine max); KRH05237.1 (Glycine max); KRH05235.1 (Glycine max); XP_014625601.1 (Glycine max); XP_014625602.1 (Glycine max). (PDF) [file pone.0212071.s010.pdf]

```
XP_007161165.1      MLLMEMTSKRKNLNPHADHSSQLYFPLWIYDHIREEKDADIEDVTEEENKIAKKMIIVAL    281
XP_007161182.1      MLLMEMASKRKNLNPYADHSSQLYFPLWIYDHIREEEDVDIEDVTEEENKIAKKMIIVAL    324
RDX99908.1          MLLMEMANKKRNLNSHAEHSSQLYFPLWIYNHLQQEKDIEMEDLTHEEKKIAKKMIIVAL    632
KRH18016.1          MLLMEMASKRKNLNPHADHSSQLYFPFWIYNQLGKETDIEMEGVTEEENKIAKKMIIVSL    587
KRH18017.1          MLLMEMASKRKNLNPHADHSSQLYFPFWIYNQLGKETDIEMEGVTEEENKIAKKMIIVSL    595
NP_001238112.1      MLLMEMASKRKNLNPHADHSSQLYFPFWIYNQLGKETDIEMEGVTEEENKIAKKMIIVSL    621
KRH18018.1          MLLMEMASKRKNLNPHADHSSQLYFPFWIYNQLGKETDIEMEGVTEEENKIAKKMIIVSL    613
RDX92217.1          MLLMEMASKRKNLNPQVEHSSQIYFPFWIYNHIGEEKDIEMEDVTEEEKKITKKMFIVAL    305
KRH18726.1          MLLMEMASKRKNLNPYAERSSQLYYPFWIYNHLVEEKDIETKDVTEEENKIAKKMIIVAL    279
KRH15824.1          MLLMEMASKRKNLNPHAERSSQLFFPFWIYNHIGDEEDIEMEDVTEEEKKIAKKMIIVAL    311
XP_014622088.1      MLLMEMASKRKNLNPHAERSSQLFFPFWIYNHIGDEEDIEMEDVTEEEKKIAKKMIIVAL    603
NP_001237957.1      MLLMEMASKRKNLNPHAERSSQLFFPFWIYNHIGDEEDIEMEDVTEEEKK----MIIVAL    562
XP_025982075.1      MLLMEMASKRKNLNPHAERSSQLFFPFWIYNHIGDEEDIEMEDVTEEEKK----MIIVAL    456
KRH05215.1          MLLMEMASKRKNLNPHAERSSQLFFPFWIYNHIGDEEDIEMEDVTEEEKK----MIIVAL    431
XP_006600115.2      MLLMEMASKRKNLNPHAERSSQLFFPFWIYNHIGDEEDIEMEDVTEEEKK----MIIVAL    579
KRH05216.1          MLLMEMASKRKNLNPHAERSSQLFFPFWIYNHIGDEEDIEMEDVTEEEKK----MIIVAL    303
KRH05221.1          MLLMEMAGKRKNLNPHAERSSQLFFPFWIYNHIRDGEDIEMEDVTEEEKKMVKKMIIVAL    456
KRH05219.1          MLLMEMAGKRKNLNPHAERSSQLFFPFWIYNHIRDGEDIEMEDVTEEEKKMVKKMIIVAL    579
XP_006601168.1      MLLMEMAGKRKNLNPHAERSSQLFFPFWIYNHIRDGEDIEMEDVTEEEKKMVKKMIIVAL    599
XP_014625496.1      MLLMEMAGKRKNLNPHAERSSQLFFPFWIYNHIRDGEDIEMEDVTEEEKKMVKKMIIVAL    476
KRH05237.1          MLLMEMASKRKNLNPHAERSSQLFFPFWIYNHIGDEEDIEMEDVTEEEKKMIKKMIIVAL    453
KRH05235.1          MLLMEMASKRKNLNPHAERSSQLFFPFWIYNHIGDEEDIEMEDVTEEEKKMIKKMIIVAL    576
XP_014625601.1      MLLMEMASKRKNLNPHAERSSQLFFPFWIYNHIGDEEDIEMEDVTEEEKKMIKKMIIVAL    585
XP_014625602.1      MLLMEMASKRKNLNPHAERSSQLFFPFWIYNHIGDEEDIEMEDVTEEEKKMIKKMIIVAL    462
                    ******:.*::***  .::***:::*:***::: .  * : :.:*.**:*    *:**:*
```
