# Supplementary material for: Genome wide association study identifies novel single nucleotide polymorphic loci and candidate genes involved in soybean sudden death syndrome resistance
Source: PLoS One. 2019 Feb 26;14(2):e0212071. doi: 10.1371/journal.pone.0212071 (PMC6391044; doi:10.1371/journal.pone.0212071)
Supplement: S9 Fig — The Glyma.14g035000 (KRH14580.1) protein identified in our study is highlighted in green. The list of highly homologous proteins to Glyma.14g035000 are: XP_007141497.1 (Phaseolus vulgaris); KOM46644.1 (Vigna angularis); RDX61322.1 (Mucuna pruriens); KRH14580.1 (Glyma.14g035000) (Glycine max); KRH73546.1 (Glycine max); AET00215.1 (Medicago truncatula); PNX72299.1 (Trifolium pratense); GAU22154.1 (Trifolium subterraneum); PKI57765.1 (Punica granatum); OWM69137.1 (Punica granatum); ONI14172.1 (Prunus persica); PQP95689.1 (Prunus yedoensis var. nudiflora); PON97614.1 (Trema orientale); POE81190.1 (Quercus suber); OIW04185.1 (Lupinus angustifolius); OIW02814.1 (Lupinus angustifolius); XP_010651515.1 (Vitis vinifera); XP_007146115.1 (Phaseolus vulgaris); KOM26446.1 (Vigna angularis); KYP52351.1 (Cajanus cajan); RDY02770.1 (Mucuna pruriens); KHN28152.1 (Glycine soja); KRH50598.1 (Glycine max); KHN14693.1 (Glycine soja); KRG89674.1 (Glycine max). (PDF) [file pone.0212071.s011.pdf]

XP_007141497.1	--HQEIKDSPLAAVSPTKKKPAPPPKSA---------	82
KOM46644.1	--HQEIKDSPRAAVSPTKNKPAPPPKST---------	80
RDX61322.1	--HQEIKESPLTTTSPAKNKAPPEAKSA---------	78
KRH14580.1	--HQEIKDSPLAATSPAKNKAPLATKSA---------	75
KRH73546.1	--SQEIKDSPLAATSPPKKKVPPATKSA---------	74
AET00215.1	EIKAEFAAPPAVTTSPVKPKAPPVNKSAST-------	88
PNX72299.1	--EQKTTAPPAVTTSPVKPKAAPVEKSA---------	80
GAU22154.1	--EQKTKAPTTVTTSPVKPKVAPVQKSA---------	80
PKI57765.1	ADKAQEGDVSAYVHSPATLKKPPGPKNT---------	92
OWM69137.1	ADKAQEGDVSAYVHSPATLKKPPGPKNT---------	92
ONI14172.1	NSPPKAHGQESSTHSPAPNKKPPTPRPSGT-------	78
PQP95689.1	NTPPKAHGQDSSTHSPAPNKKPPTPRPSGT-------	78
PON97614.1	NQQNTTQQDASVGNSPAPEKKPPSSRST---------	72
POE81190.1	SHTATHQDASVSTRCPATPKKPPAVKPASACKAEDQC	88
OIW04185.1	KQTPPTNPPPPAT-----TNKPPPAKSH---------	79
OIW02814.1	KDAPTTTHPSPAT-----VKKPPPAKSA---------	70
XP_010651515.1	-DPPTTAQPAPATHFPAPGSKPPAGRAAGV-------	81
XP_007146115.1	GEKNSVATPSPATAC---KKPPPPGKPA---------	74
KOM26446.1	EEKNSVTTPSPANTS---KKLPPPQGKSA--------	75
KYP52351.1	QGRQTTLTTPPATAT---KKPPPPEKST---------	95
RDY02770.1	QERETTLITPPPVTA---TKKPPPGKST---------	75
KHN28152.1	QGRQ---IPSPATAA---KKSLPPGKST---------	73
KRH50598.1	QGRQ---IPSPATAA---KKSLPPGKST---------	126
KHN14693.1	QERQ---APSPVTTA---KKLPPPGKST---------	74
KRG89674.1	QERQ---APSPVTTA---KKPPPPGKST---------	74
