# Supplementary material for: Genome wide association study identifies novel single nucleotide polymorphic loci and candidate genes involved in soybean sudden death syndrome resistance
Source: PLoS One. 2019 Feb 26;14(2):e0212071. doi: 10.1371/journal.pone.0212071 (PMC6391044; doi:10.1371/journal.pone.0212071)
Supplement: S10 Fig — The Glyma.01G222900.1 protein identified in our study is highlighted in green. The list of highly homologous proteins to Glyma.01G222900.1 are: XP_006574309.1 (Glycine max); XP_006591523.1 (Glycine max). (PDF) [file pone.0212071.s012.pdf]

```
XP_006574309.1          MAKSLSSPVANKSSMTTEEVIAMFPSRKEEKE-SSKCLVYALVVFVAILFIWLVFASIVL 59
Glyma.01G222900.1       MAKSLSSPVANKSSMTTEEVIAMFPSRKEEKE-SSKCLVYALVVFVAILFIWLVFASIVL 59
XP_006591523.1          MAKSLSSPSPNKYNMTTEEVITMFPSRKEEKQSSSKCLVYALVVLVAILFIWLVFASIVL 60
                        ********  ** .*******:*********: ***********:***************
```
