# Supplementary material for: Genome wide association study identifies novel single nucleotide polymorphic loci and candidate genes involved in soybean sudden death syndrome resistance
Source: PLoS One. 2019 Feb 26;14(2):e0212071. doi: 10.1371/journal.pone.0212071 (PMC6391044; doi:10.1371/journal.pone.0212071)
Supplement: S11 Fig — The Glyma.10G058700.1 (KRH32551.1) protein identified in our study is highlighted in green. The list of highly homologous proteins to Glyma.10G058700.1 are: XP_016651648.1 (Prunus mume); XP_018814810.1 (Juglans regia); XP_008241451.1 (Prunus mume); XP_024022989.1 (Morus notabilis); POO01767.1 (Trema orientale); PON49032.1 (Parasponia andersonii); XP_017251878.1 (Daucus carota subsp. sativus); PKI57622.1 (Punica granatum); XP_010039059.1 (Eucalyptus grandis); KRH32551.1 (Glycine max); XP_025979781.1 (Glycine max); XP_017413005.1 (Vigna angularis); XP_014511918.1 (Vigna radiata var. radiata); XP_006595362.1 (Glycine max); RCW19085.1 (Glycine max); XP_007144374.1 (Phaseolus vulgaris); KHN48245.1 (Glycine soja); XP_020239895.1 (Cajanus cajan); KYP41914.1 (Cajanus cajan); XP_004515935.1 (Cicer arietinum); XP_024633997.1 (Medicago truncatula); XP_004494902.1 (Cicer arietinum); XP_007162785.1 (Phaseolus vulgaris); XP_020211654.1 (Cajanus cajan); KYP70913.1 (Cajanus cajan). (PDF) [file pone.0212071.s013.pdf]

```
XP_016651648.1       STIIGVHFGHVLIQMKGHPARLKHWIPAGCALLVLGIILHFTHAIPSNKQLYTFSYVCIT    280
XP_018814810.1       SGTIGIHYGHVLIHFKGHAERLRQWVSMGFILLTVAIILHFTDAIPMNKQLYSFSYVCFT    397
XP_008241451.1       SGTIGIHYGHVLIHFKGHSERLKQWVSMGFILIVMAIILHFTDAIPINKQLYSFSYVCFT    400
XP_024022989.1       SGTIGIHYGHVLIHFKGHSERLKHWVSMGFGLLIIGIILHFTDAIPINKQLYSFSYVCFT    395
POO01767.1           SGTIGIHYGHVLIHFKGHSERLKQWLSMGFGLLIIGIILHFTDAIPINKQLYSFSYVCFT    405
PON49032.1           SGTIGIHYGHVLIHFKGHSERLKQWLSMGFGLLIIGIILHFTDAIPINKQLYSFSYVCFT    405
XP_017251878.1       SGTIGIHYGHVLIHFKGHAERLKQWVSMGFGLLILAIILHFTDAIPINKQLYSFSYVCFT    390
PKI57622.1           SGTIGIHYGHVLVHFKGHAERLKQWVSMGLCLLVVAIILHFTDAIPINKQLYSFSYVCFT    132
XP_010039059.1       SGTIGIHYGHVLIHFKGHSERLKQWVSMGLCLLIVAIILHFTDAIPINKQLYSFSYVCFT    388
KRH32551.1           SGTIGIHYGHVLIHFKGHSERLKQWLLMGFLLLTLGLMLHFTEAIPINKQLYSFSYVCFT    243
XP_025979781.1       SGTIGIHYGHVLIHFKGHSERLKQWLLMGFLLLTLGLMLHFTEAIPINKQLYSFSYVCFT    247
XP_017413005.1       SGVIGIHYGHVLIHFKGHSERLKQWLTLGLVLFTIGLILHFTDAIPINKQLYSFSYVCFT    363
XP_014511918.1       SGVIGIHYGHVLIHFKGHSERLKQWLTLGLVLFTVGLILHFTDAIPINKQLYSFSYVCFT    360
XP_006595362.1       SGTIGIHYGHVLIHFKGHFERLKQWLSMGFVLLTLGLILHFTDAIPINKQLYSFSYVCFT    372
RCW19085.1           SGTIGIHYGHVLIHFKGHFERLKQWLSMGFVLLTLGLILHFTDAIPINKQLYSFSYVCFT    345
XP_007144374.1       SGIIGIHYGHVLIHFKGHSERLKQWLSLGFFLLIIGIILHFTDAIPINKQLYSFSYVCFT    390
KHN48245.1           SGTIGIHYGHVLIHFKGHFERLKQWLSMGFVLLTLGLILHFTDAIPINKQLYSFSYVCFT    392
XP_020239895.1       SGTIGIHYGHVLIHFKGHSERLKQWLSMGFVLLILGIILHFTNAIPINKQLYSFSYVCFT    387
KYP41914.1           SGTIGIHYGHVLIHFKGHSERLKQWLSMGFVLLILGIILHFTNAIPINKQLYSFSYVCFT    381
XP_004515935.1       SGTIGIHYGHVLIHFKGHSERLKQWLSMGFVLFILGIILHFTDAIPINKQLYSISYVCFT    370
XP_024633997.1       SGTIGIHYGHVLIHFKGHSERLKQWLSMGVVLFILGIILHFTNAIPINKQLYSISYVCFT    387
XP_004494902.1       SGTIGIHYGHVLIHFKGHSERLKQWVSMGFVLLTIAIILHFTNAIPINKQLYSISYVCLT    368
XP_007162785.1       SGTIGIHYGHVLIHFKGHSERLKQWVSMGFVLLIIAIILHFTDAIPINKQLYSFSYVCFT    376
XP_020211654.1       SGTIGIHYGHVLIHFKGHSERLKQWVSMGFVLLIIAIILHFTDAIPLNKQLYSFSYVCFT    388
KYP70913.1           SGTIGIHYGHVLIHFKGHSERLKQWVSMGFVLLIIAIILHFTDAIPLNKQLYSFSYVCFT    390
                     *  **:*:****:::***  **::*:  *  *: :.::****.*** *****::****:*
```
